# A dedicated partitioning machinery ensures faithful inheritance of the apicoplast in ***Toxoplasma gondii***

**DOI:** 10.1038/s41467-026-78103-w

**Published:** 2026-10-05

**Authors:** Garret C. Eichlin, Prince Oppong, Hallie R. Hayne, Pamodya Pamunuwa, Benjamin Chartier, Eranthie Weerapana, Marc-Jan Gubbels, Klemens Engelberg

**Affiliations:** 1 Department of Biology, Boston College, Chestnut Hill, MA USA; 2 Department of Chemistry, Boston College, Chestnut Hill, MA USA

**Keywords:** Parasite biology, Parasitology

## Abstract

*Toxoplasma gondii* tachyzoites divide by endodyogeny, an internal budding process that requires precise machinery for equal partitioning of single-copy organelles into daughter cells. The essential apicoplast is partitioned by temporal association with the centrosome, which is orchestrating various cell division processes. However, the identity of the molecular infrastructure facilitating anchoring of the apicoplast to the centrosome is still unknown. Here, we describe the apicoplast partitioning protein 1 (APP1), which first assembles in a specific basal complex (BC) section and then localizes in close proximity to the apicoplast. Conditional APP1 knockdown results in a specific apicoplast partitioning defect. Reciprocally, upon apicoplast loss through ATG8-depletion, APP1 remains associated with the cytoskeleton, consolidating a role in apicoplast anchoring to the cell division infrastructure. Using APP1 as bait in a proximity biotinylation screen, we identify APP3, which positions APP1 to the specific site on the BC, but has overall a more pleiotropic role in coordinating cell division. In summary, APPs are functional connectors between the apicoplast and the daughter cell scaffold (DCS) critical for apicoplast inheritance. Together our findings provide novel mechanistic insights into this unique aspect of apicomplexan organelle biology.

## Abstract (web summary)

Many apicomplexan parasites possess an essential apicoplast organelle. Here, Eichlin et al. identify APP proteins in *T. gondii*, which are critical for anchoring the apicoplast to the centrosome and the cytoskeleton, thereby facilitating its inheritance during cell division.

## Introduction

Faithful partitioning of eukaryotic organelles that cannot be generated de novo is essential for successful cell division. Two principally different strategies exist: 1. random, stochastic partitioning based on multi-copy or fragmented organelles; 2. active partitioning of single-copy organelles. The latter is typically seen in smaller, single-celled eukaryotes like yeast and algae^1,2^. For active partitioning a tight coupling with the cell division machinery is required wherein the cytoskeleton needs to align, anchor and/or position the organelles together with anchors and connectors between the organelle and cell division machinery.

Apicomplexan parasites, including *Toxoplasma gondii* and *Plasmodium spp*., cause major human diseases such as toxoplasmosis and malaria, respectively. These pathogenic protozoa harbor a unique organelle, the apicoplast, a four-membrane-surroundeded organelle acquired by secondary endosymbiosis of a chloroplast containing red alga^3^. Although photosynthetic capacity was lost, the apicoplast supports vital metabolic pathways such as the generation of isoprenoids, fatty acids, and heme^4,5^.

The asexual tachyzoite stage of *T. gondii* divides by an internal budding process called endodyogeny. Single copy organelles like the apicoplast, Golgi apparatus, and the mitochondrion require a precise machinery to ensure that each daughter cell receives exactly one organelle. Organelle dynamics during cell division^6^ revealed that the mitochondrion is the very last organelle to be partitioned through a parasite-specific cytoskeleton scaffold anchor^7^, whereas the Golgi apparatus and apicoplast are anchored to the centrosome^8–10^. The centrosome is the spatiotemporal organizational hub of *T. gondii* cell division^11,12^, but the identity of the molecular infrastructure responsible for anchoring the single copy organelles to the centrosome is still unknown.

A key factor in apicoplast partitioning is the autophagy machinery through a non-canonical function. Notably the ATG8-ATG5-VPS15-SNAP29 complex acts on the apicoplast through phosphoinsitide (PI) metabolism^13–16^. The apicoplast outer membrane is enriched in PI3P and overexpression of a PI3P sensing construct (DD-FYVE) leads to apicoplast loss^15,17^. Together with the essential role for lipid kinases in apicoplast homeostasis^18^, the various ATG-pathway mediated perturbations of PI3P traffic support a role in apicoplast biogenesis, which represents a largely indirect effect on apicoplast inheritance^4^.

Along a similar mechanism, another factor needed for biogenesis is the actin-myosin machinery. Myosin F (MyoF) is needed to traffic vesicles from the endoplasmic reticulum to the apicoplast. Without MyoF, the apicoplast does not elongate during division and fails to become anchored to the centrosomes, which facilitate its partitioning^19,20^. More recently, a membrane-associated YGGT domain-containing apicoplast protein (YCAP) was shown to participate in apicoplast inheritance^21^. Given domain structure and putative orthologous functions, it is possible that YCAP is used for importing nuclear encoded apicoplast (NEAT) proteins or might be involved in the fission/division of the organelle rather than directly in partitioning. Furthermore, an apicoplast partitioning defect was reported upon depletion of MORN1, a scaffolding protein in the centrocone (spindle pole), basal complex (BC: contractile ring), and in a ring structure at the apical end of the IMC, but the details on this mechanism are unknown^22,23^.

In this study, we describe the apicoplast partitioning proteins (APPs), of which APP1 is a 2300 amino acid (aa) protein conserved among the Coccidia. During cell division, APP1 first accumulates on the BC, followed by a shift to a region on the daughter cell scaffold (DCS) in close proximity to the apicoplast and the centrosome. Furthermore, conditional knockdown resulted in an apicoplast partitioning defect. Via BioID we identified APP3, a large 4037 aa protein that facilitates APP1 localization to one side of the forming daughter cells. In summary, our findings establish APPs as critical cytoskeleton-anchored facilitators of apicoplast inheritance, providing a key new insight into the innovative, parasite-specific biology associated with this unique organelle.

## Results

### APP1 discovery and description

**Fig. 1:**
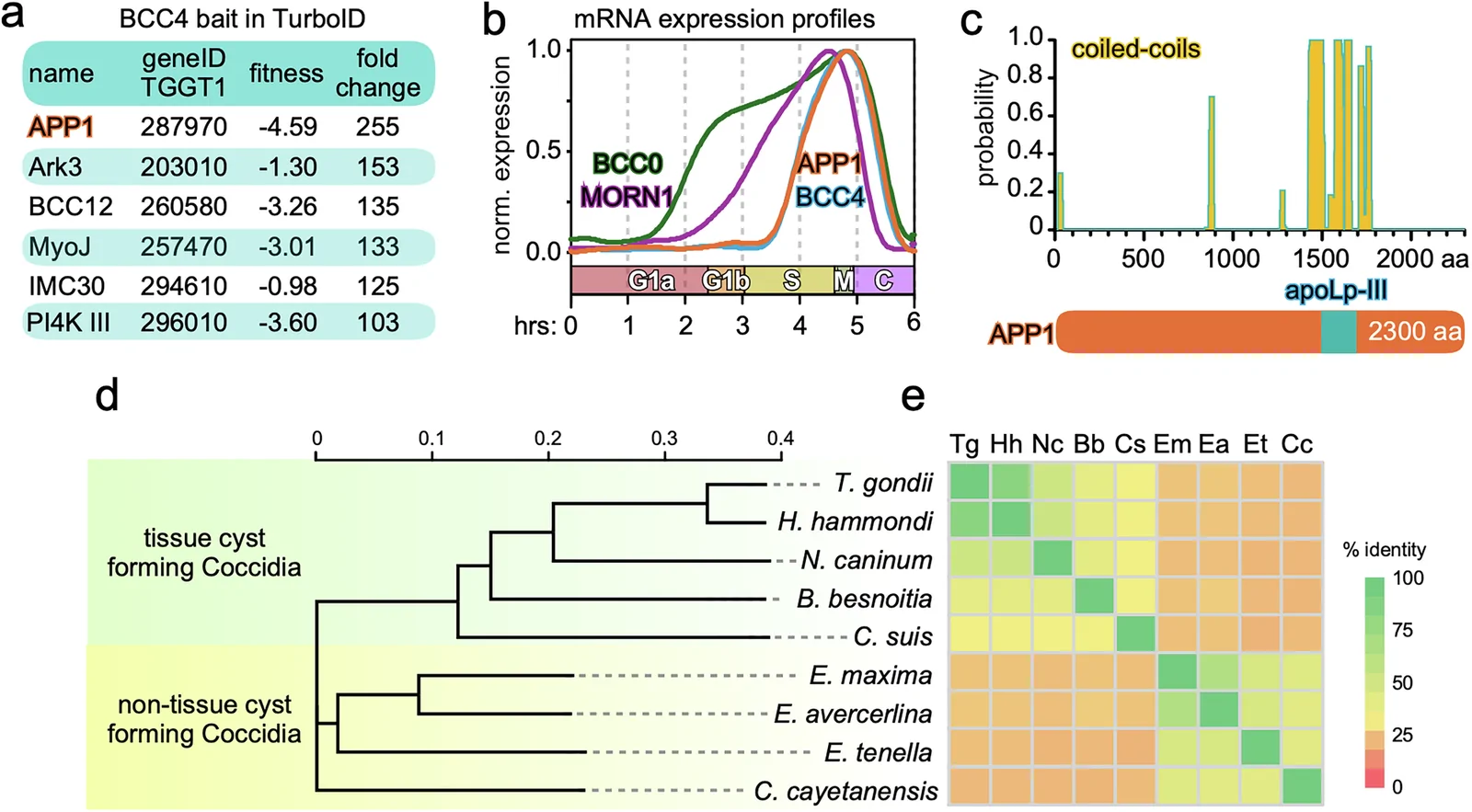
APP1 features. **a** Identification of APP1 by TurboID using BCC4 as bait. Lytic cycle fitness score from^27^; fold change represents enrichment in intracellular vs. extracellular parasites (hits >100-fold enriched are shown). BCC12 is a BC-residing protein. **b** Normalized gene expression profiles based on pseudo time from scRNA-seq data^60^ show APP1 and BC components BCC4 (bait), MORN1 and BCC0. **c** APP1 sequence analysis identified coiled-coil domains (sliding window size of 28 is plotted^61^) and detected a putative Apolipophorin-III (apo-LpIII) domain by SMART protein database^62^ search (SCOP domain d1eq1a_ with an E-value of 5E-4^63^). **d** APP1 is conserved among Coccidian parasites only. *Hammondia hammondi* HHA_287970; *Neospora caninum* NCLIV_040630; *Besnoitia besnoiti* BESB_047830; *Cystoisospora suis* CSUI_004882; *Eimeria maxima* EMWEY_00032490; *Eimeria acervulina* EAH_00023840; *Eimeria tenella* ETH2_1133900; *Cyclospora cayetanensis* cyc_01346. **e** APP1 percent identity matrix in orthologs.

In our pursuit to map the BC proteome by proximity biotinylation^24^, the top hit using BCC4 as bait was an unknown protein, TGGT1_287970 (Fig. 1a). We named this protein APP1 based on the work presented here. Hit specificity was supported by the nearly identical mRNA expression profiles of BCC4 and APP1 throughout the cell division cycle, which differs from genes with earlier functions in initiating cell division like BCC0 and MORN1 (Fig. 1b). At the primary sequence level, APP1 is a large, 2300 amino acid protein in which we could identify some coiled-coil regions, as well as detected a putative Apolipoprotein L-III (apoLp-III) domain suggestive of lipid binding (Fig. 1c). Next, we asked whether APP1 was conserved across the Apicomplexa or even beyond this phylum. APP1 orthologs with high levels of similarity were detected throughout the Coccidia, comprising *T. gondii* and its relatives, but no orthologs were identified in more distantly related Apicomplexa or their free-living relatives (Fig. 1d). Even within the Coccidia the percentage identity was low, in general below 50%, and fell into two blocks along the tissue cyst forming vs. non-tissue cyst forming Coccidia (Fig. 1e). Overall, the APP1 expression profile overlaps with BCC4 and APP1 is conserved across the Coccidia.

### APP1 localizes in close apposition to the centrosome and is confined to one side of the forming daughter bud

**Fig. 2:**
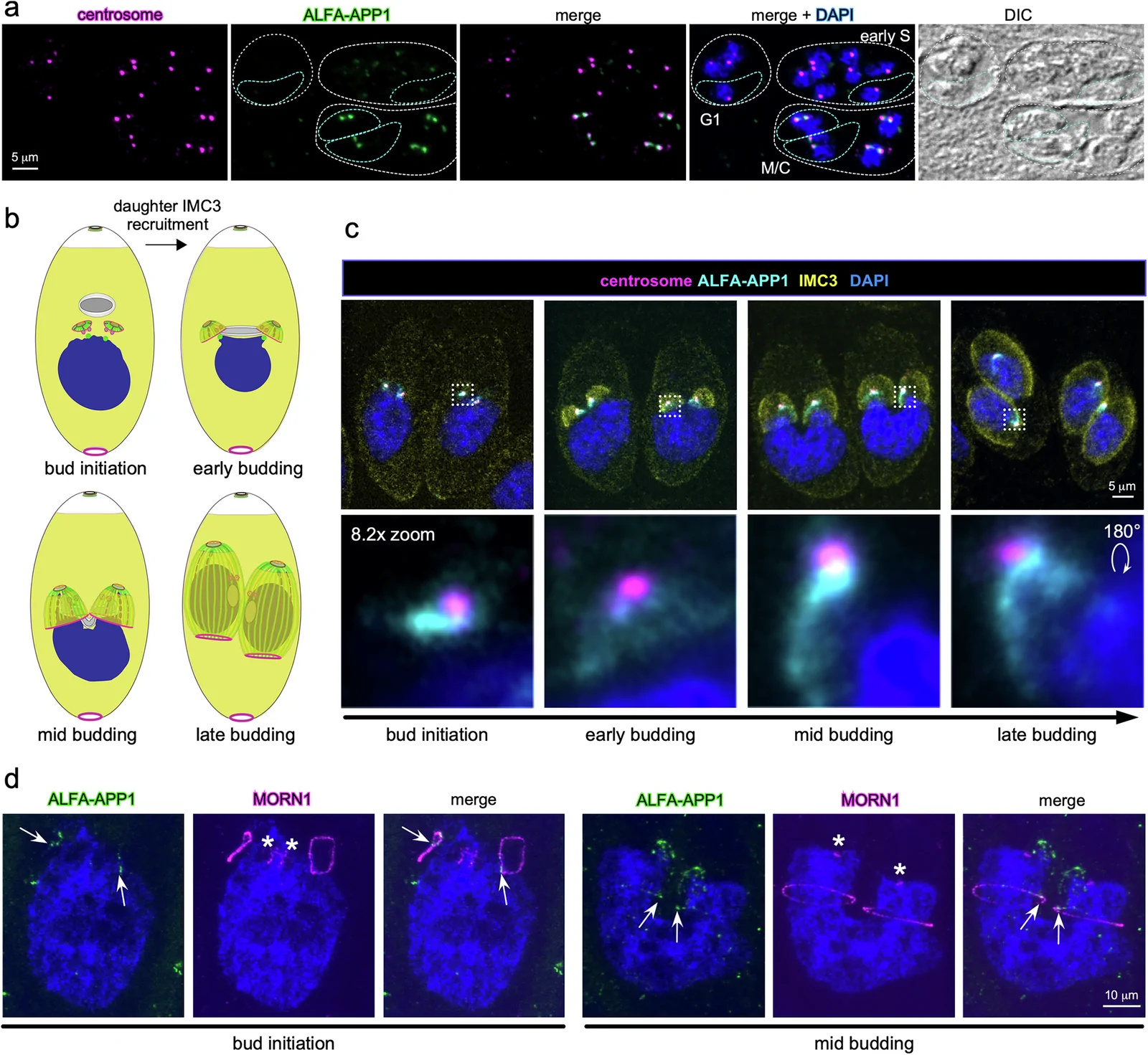
APP1 localizes in between the centrosome and the daughter basal complex. **a** APP1 appears near the centrosome at early S phase, before duplicated centrosomes are visible, increases in signal strength during M/C phase, but is undetectable in G1. White and blue dotted lines outline parasitophorous vacuoles and select individual parasites, respectively. Images collected by conventional IFA and wide field microscopy (Zeiss Z2+Apoptome). **b** Schematic of *T. gondii* endodyogeny, with most of the maternal structures omitted. Two daughter cells form apical to the centrosome (magenta: centrioles of the outer core, green: spindle pole of the inner core) in the cytosol of the mother. Daughter cells predominantly grow in the apical-to-basal direction and are capped by the BC (pink) at their posterior ends. Note that recruitment of IMC3 to the forming buds unfolds after their initiation. Yellow: IMC3, gray: apicoplast, orange: SFA fiber, green: microtubules/tubulin fibers, brown: APR, blue: nucleus, and purple: sutures (with two to three of the five to six sutures shown). **c** APP1 localizes basal to the centrosome and is confined to only one side of the forming daughter cells. Centrosome visualized via Centrin (Cen20H5) antiserum. Inlays are 8.2x zoom of dotted areas. PS-ExM with Airyscan imaging. **d** APP1 is deposited on the daughter BC (highlighted by MORN1), at bud initiation (arrow). At the mid-budding stage, APP1 is localized to only one side of the forming daughter and still connected to the daughter BC (arrow). Asterisks mark MORN1 in the centrocone (spindle pole). DAPI stains DNA. Images were collected by pan-ExM and Airyscan microscope.

Since APP1 was not detected in the HyperLOPIT organellar proteome of *T. gondii*^25^, we tagged the encoding gene at its 5’end with an ALFA epitope tag (Supplementary Fig. 1b) to assess its localization. We then co-stained ALFA-APP1 with compartment-specific markers. Spatially, we observed a close apposition between APP1 and the centrosome (Fig. 2a). Temporally, APP1 appears before duplicated centrosomes are detectable by IFA (early S marked vacuole in Fig. 2a, schematic summary of *T. gondii* endodyogeny in Fig. 2b), increases in signal intensity throughout M/C phase, and then disappears upon completion of cell division (G1). This protein level pattern closely aligns with the mRNA expression profile, which, together with the close centrosome apposition, supports a role in cell division.

To enhance the resolution of APP1’s localization, we pivoted to  pre-gelation staining expansion microscopy (PS-ExM^26^, Fig. 2c) and iterative expansion microscopy (pan-ExM, Fig. 2d). We first co-stained for the centrosome and the DCS marker IMC3. This revealed that APP1 appears after the centrosomes duplicate, but before IMC3 is recruited to the early DCS (Fig. 2c, bud initiation). When the bud assembles, APP1 stretches out in a bended structure from the basal end of the daughter bud toward the centrosome, and eventually loses the connection to the BC in the late budding stage (Fig. 2c). Co-staining of APP1 with the BC marker MORN1 at even higher pan-ExM resolution showed that APP1 initially associates with the BC at the basal end of the early DCS, when the mitotic spindle just formed (Fig. 2d, bud initiation). Mid budding, when the nucleus has started its transition into the nascent buds, APP1 is seen in a patchy, cone-shaped pattern along the two lobes of the dividing nucleus (Fig. 2d, mid budding). At this stage, APP1 is in contact with the BC (Fig. 2d, mid budding, arrows) and marks a structure that is extending apically, beyond the spindle poles marked by MORN1 (Fig. 2d, mid budding, asterisks). Staining of total protein with NHS-ester, which visualizes protein density, revealed that the association of APP1 with the BC occurs on the side of the forming DCS that harbors the centrosome (Supplementary Fig. 2, Supplementary Movie 1). This further confirmed that APP1 does not localize to the entire BC, as it exhibits a sickle-shaped signal instead of the continuous ring expected for a BC localization (Supplementary Movie 1).

Overall, our data reveal that APP1 localization is dynamic, with initial recruitment to a BC section facing the centrosomes early in the budding cycle before IMC3 decorates the forming buds. From this initial BC localization, the signal stretches out, staying anchored to the BC and the centrosome until the BC connection is lost in the late budding stage.

### APP1 is essential for apicoplast partitioning

**Fig. 3:**
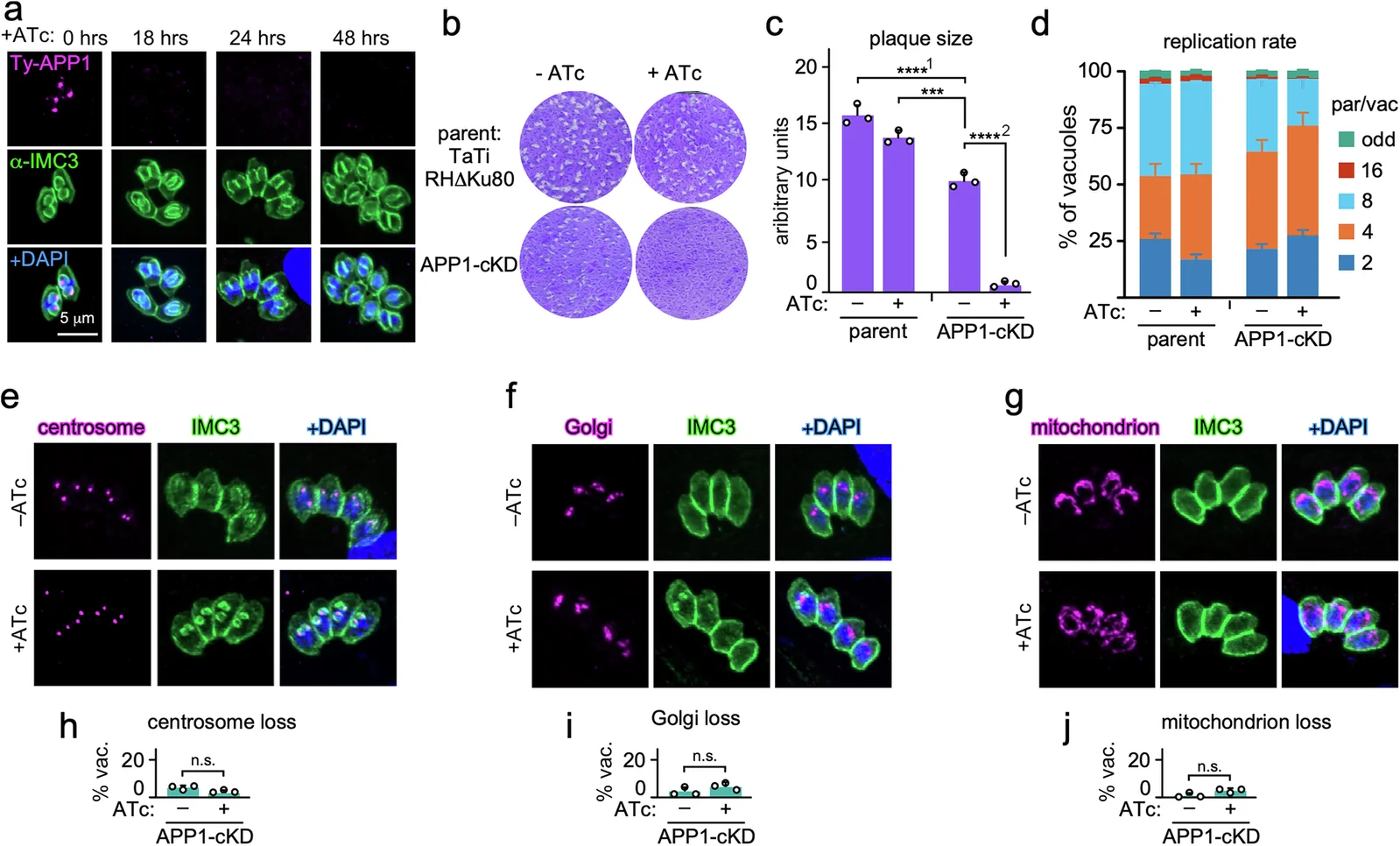
APP1 is critical for proliferation, but does not affect replication rate or partitioning of the centrosome, the Golgi apparatus and the mitochondrion. **a** Conditional knock-down (cKD) of Ty-APP1 under control of the TetO7sag4 promoter suppresses APP1 within 18 hrs to undetectable levels by immunofluorescence (Z2). **b** Plaque assays (9 days) of APP1 depleted parasites show a severe fitness effect. **c** Quantification of plaque sizes shown in (**b**). Data represent mean values +std of *n* = 3 biological replicates; one-way ANOVA with Tukey’s honestly significance difference (HSD) post hoc test.*** *p* = 0.000386; ****^1^ *p* = 1.81 × 10^−5^, ****^2^ *p* = 5.49 × 10^−7^. **d** APP1-cKD parasite replication rate is normal. The number of parasites per vacuole was counted 24 hrs ±ATc addition by IFA using IMC3 antiserum and DAPI stains on a widefield DeltaVision microscope. Average of *n* = 3+std biological replicates, with 100 vacuoles quantified, is plotted. The ‘odd’ bin collects all vacuoles with non-geometric expansion numbers (1 par/vac were not counted). **e–g** APP1-cKD (24 hrs +ATc) does not interfere with partitioning of *T. gondii* organelles, like the centrosome (highlighted via Centrin (Cen20H5) antiserum) (**e**), the single copy Golgi apparatus (stained with TgSRTL antiserum) (**f**) or the single copy mitochondrion (stained with F_1_B-ATPase antiserum) (**g**). Representative images of quantifications presented in (**h–j**). IMC3 antiserum highlights the cytoskeleton. DAPI stains DNA. Images collected by conventional IFA and wide field microscopy (Zeiss Z2+Apoptome). **h–j** Quantification of organelle loss. The abnormal phenotypes were defined as the absence of respective organelle in one or more of the daughter cells sharing a vacuole. Data represent mean values +std of *n* = 3 biological replicates, with 50 vacuoles quantified for each replicate; Two-tailed Welch’s *t-*test: n.s. non-significant.

APP1’s severe fitness score of −4.59 hints at an essential function in the lytic cycle^27^. To define its function, we generated a conditional knockdown by using the Tet-Transactivation system (TATi), which can be induced with anhydrous tetracycline (ATc)^28^ (Supplementary Fig. 1a). Upon ATc application, APP1 becomes undetectable after 18 hrs (Fig. 3a). We did not observe morphological changes, either in the mother or daughter tubulin cytoskeleton or the daughter BC (Supplementary Fig. 3). By plaque assay, we however observed a severe 92.4% (± 2.3 std) reduction in plaque size (compared to APP1-cKD in absence of ATc), consistent with a critical role for APP1 in proliferation (Fig. 3b, c). We note that even without ATc, plaque size is reduced compared to the parent line (36.8% ± 6.6 std reduction), suggesting that changes in APP1 expression level and/or timing due to promoter replacement are critical for the parasite. To further probe into this, we analyzed the replication rate of the parasite following 24 hrs of ATc treatment, but we observed no statistical differences (Fig. 3d).

**Fig. 4:**
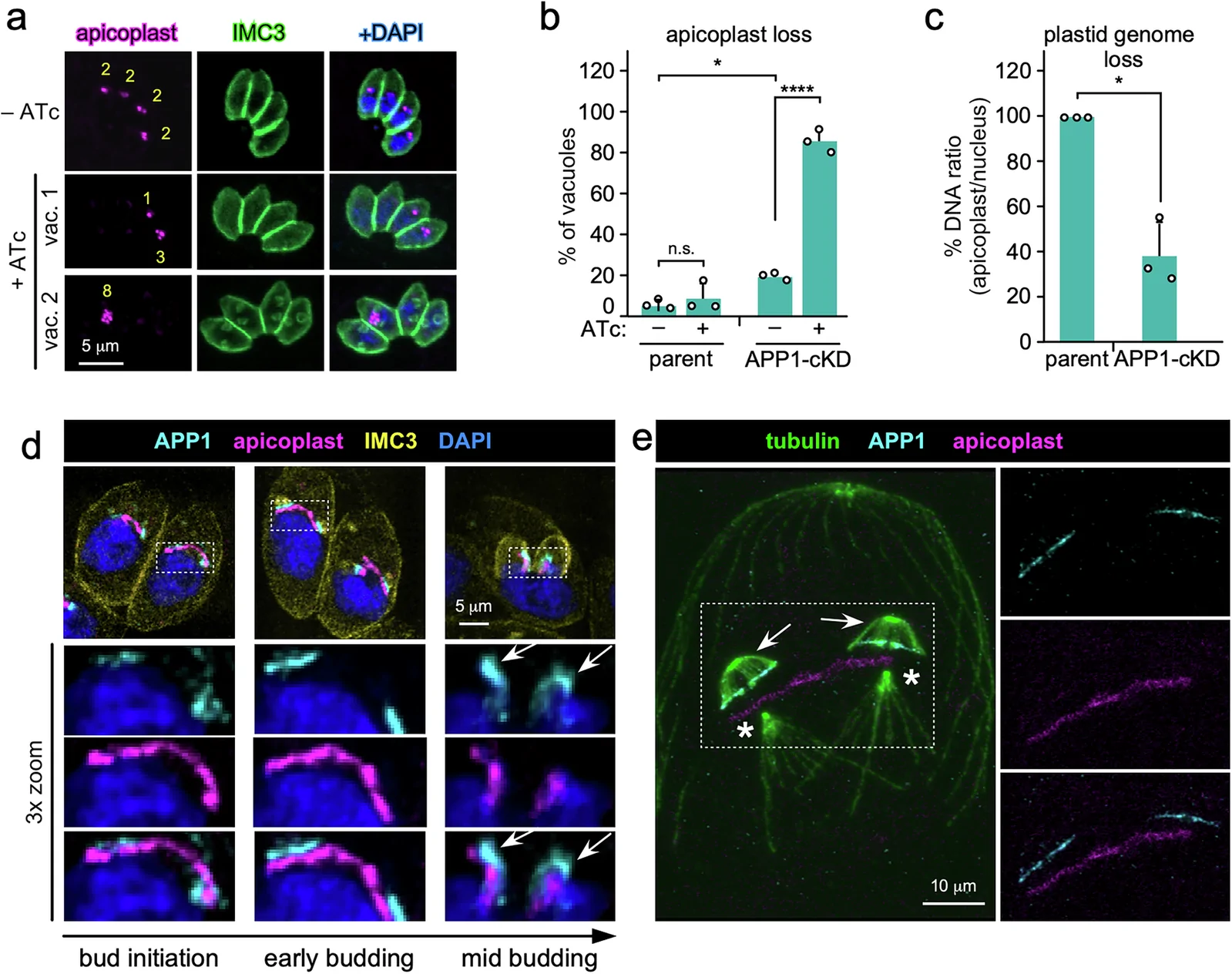
APP1 is essential for apicoplast partitioning. **a** APP1-cKD disrupts apicoplast partitioning (24 hrs +ATc). The apicoplast was stained with streptavidin, which recognized endogenously biotinylated, apicoplast proteins. Two representative vacuoles are shown; #1 is in G1; #2 just started budding. Yellow numbers report the number of streptavidin foci. Images collected by conventional IFA and wide field microscopy (Zeiss Z2+Apotome). **b** Quantification of apicoplast loss, based on microscopy shown in (**a**). The abnormal apicoplast phenotype was defined as the absence of an apicoplast in one or more parasites inside a vacuole. Data represent mean values +std of *n* = 3 biological replicates; one-way ANOVA with Tukey’s HSD post hoc test: * *p* = 0.0487; **** *p* = 4.55 × 10^−10^; n.s. = non significant. 100 vacuoles were quantified per replicate, the parent is RH∆Ku80-TATi-∆HX. **c** Apicoplast genome loss monitored by qPCR. Genomic DNA from parent and APP1-cKD lines was collected at 0 and 72 hrs post ATc addition. Fold change of the apicoplast genome in relation to the nuclear genome was calculated using the 2^-∆∆CT^ method^54^. Data represent mean values +std of *n* = 3 biological replicates. Two-tailed Welch’s *t-*test, *p = 0.011674. The parent is RH∆Ku80-TATi-∆HX, which was normalized to 100%. **d** APP1 dynamics compared to the apicoplast during cell division. At bud initiation, APP1 localizes to the elongated edges of the apicoplast. Early in budding, APP1 localizes to the basal ends of the daughter IMC3 signal, directly above the leading edges of the apicoplast. Mid-budding, APP1 is located between the apicoplast and the daughter bud IMC, with a signal accumulation apical to the leading edge of the apicoplast (arrows). APP1 visualized with ALFA antiserum, apicoplast visualized via streptavidin. DAPI stains DNA; IMC3 is highlighted via IMC3 antiserum. PS-ExM with Airyscan microscopy. **e** Pan-ExM of early budding parasites, highlighting the spatial relation of the DCS (α-Tubulin antiserum, arrows), APP1, and the apicoplast. Individual channels for boxed area are shown on the right. APP1 is visualized with ALFA antiserum; the apicoplast is visualized via streptavidin. Asterisks mark the spindle poles (centrosome inner cores). Image acquired via Airyscan microscopy. For a 3D projection, see Supplementary Movie 2.

Next, we performed IFAs with organelle-specific markers. We observed normal appearances of the centrosome, the Golgi, and the mitochondrion upon APP1 knockdown (Fig. 3e-j). However, APP1-depletion results in defective apicoplast partitioning (Fig. 4a, b), and we observed parasites lacking the organelle in 85.3% (± 5.5 std) of vacuoles when staining with streptavidin, which binds to biotinylated acetyl-CoA carboxylase 1 and 2 (ACC1 and ACC2) in the apicoplast lumen^29^ (Fig. 4a, b). Moreover, the number of streptavidin foci did add up to the expected number of apicoplasts per vacuole, although incorrectly partitioned between individual parasites (Fig. 4a, +ATc). This suggests that the absence of APP1 does not interfere with apicoplast fission. We further validated apicoplast loss via qPCR, where we observed a 41.17% (± 12.9 std) reduction of the apicoplast genome in APP1 knockdown parasites after 72 hrs of ATc treatment, which corresponds to a 62.2% (± 14.2 std) apicoplast reduction compared to the parent line TATi∆HX (Fig. 4c). This indicates that over time parasites lose the apicoplast, which prompted us to examine APP1 localization dynamics in relation to the apicoplast. At bud initiation, APP1 is in close proximity to the edges of the elongated apicoplast (Fig. 4d, bud initiation). With the progress of division and emergence of IMC3-positive daughter buds, APP1 localizes directly between the DCS basal ends and the apicoplast (Fig. 4d, early budding). At the mid-budding stage, APP1 was present between the apicoplast, which had completed fission, and the daughter IMC, thereby decorating the entirety of the organelle (Fig. 4d, mid budding). Notably, APP1 foci apical to the apicoplast indicated that APP1 is predominantly localized to the leading edge of the organelle during its translocation into the forming daughter buds (Fig. 4d, arrows).

We further investigated the early budding stages by pan-ExM, which revealed that APP1 is sharply focused at the basal ends of the DCS (Fig. 4e and Supplementary Movie 2), consistent with its above reported localization to the BC (Fig. 2d). At this stage, the elongated apicoplast is located between the forming daughter cells and the spindle poles (Fig. 4e, asterisks), with the edges of the organelle being positioned under the two forming daughter cells. We noticed a considerable gap between the apicoplast–highlighted by streptavidin–and the APP1 signal, but note that streptavidin marks the lumen of the apicoplast and fails to highlight the four membranes that surround the organelle.

Taken together, high-resolution imaging in combination with our functional dissection indicates that APP1 is dedicated to apicoplast anchoring in the forming DCS. To establish if APP1 is in immediate contact with the apicoplast membranes, we next investigated the apicoplast-localized protein ATG8.

### Mechanistic dissection of APP1’s role in apicoplast partitioning

**Fig. 5:**
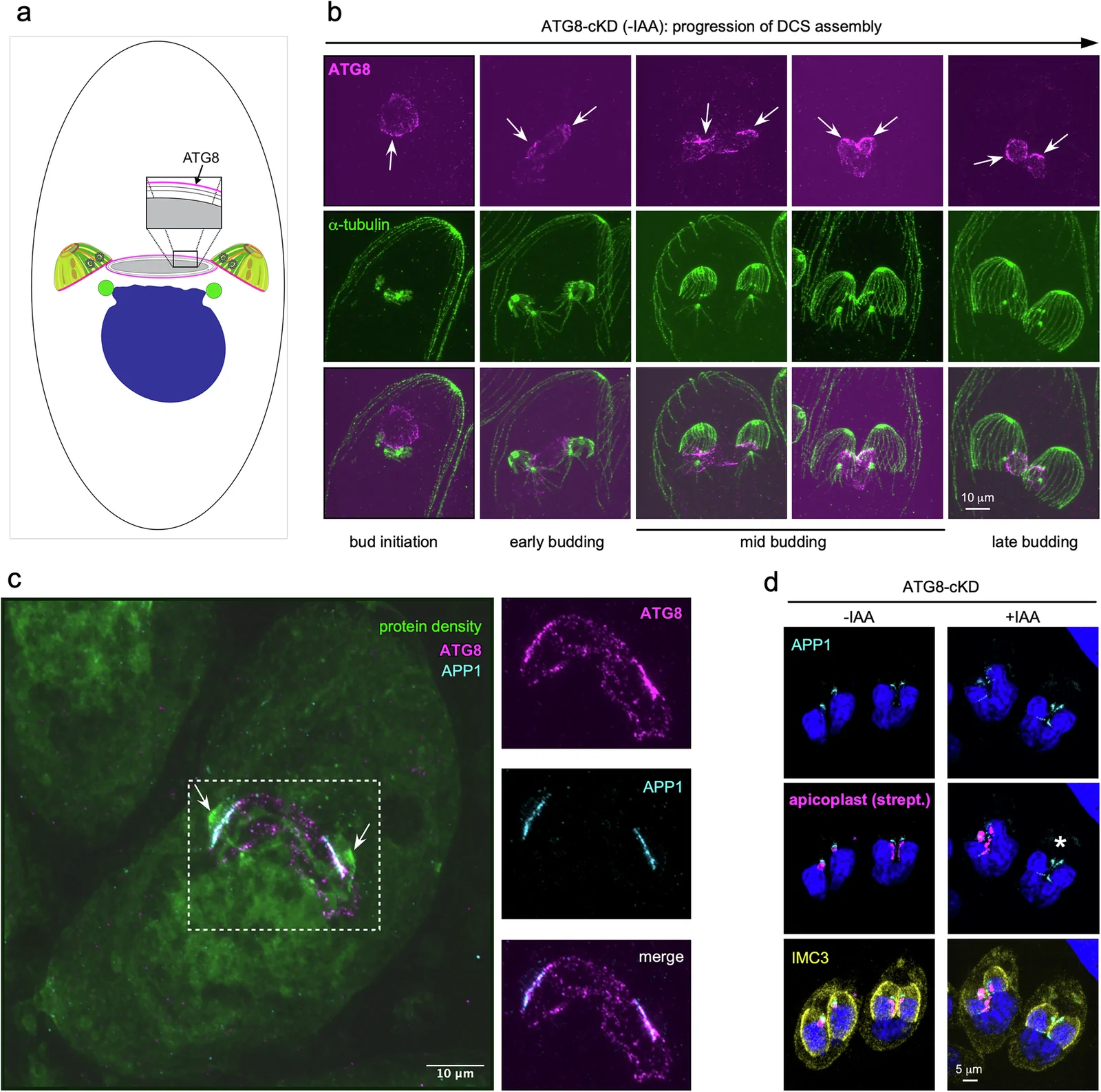
Upon plastid loss, APP1 remains localized to the DCS. **a** Schematic of a dividing tachyzoite highlighting the localization of ATG8 on the outer membrane of the apicoplast as previously described in ref. ^16^. **b** Time course of ATG8 localization throughout DCS assembly in the V5-mAID-ATG8 parasite line. ATG8 resides in the apicoplast outer membrane. Images collected by pan-ExM and Airyscan microscopy. Arrows indicate ATG8 accumulation overlapping with daughter cells. **c** Total protein staining (protein density visualized via NHS-ester (green)) of a dividing tachyzoite, highlighting the apicoplast (V5-mAID-ATG8, magenta) and ALFA-APP1 (cyan). Two daughter cells are initiated (arrows). Individual channels for dotted area are shown on the right. Image collected by pan-ExM and Airyscan imaging. For a 3D projection see Supplementary Movie 4. **d** ATG8 depletion (24 hrs +IAA) results in apicoplast loss (streptavidin; asterisk), but APP1 is retained on the DCS. Images collected by PS-ExM and Airyscan microscope.

ATG8 localizes to the outer apicoplast membrane^16^, which is different from the luminal, biotinylated markers visualized via streptavidin staining. Therefore, we tested ATG8 in pan-ExM throughout cell division (Fig. 5a), and relative to APP1 (Fig. 5b). In general, ATG8 has a much plumper appearance than the luminal proteins highlighted by streptavidin (Supplementary Fig 4, Supplementary Movie 3). Moreover, ATG8 is enriched in the areas where the apicoplast is in contact with the forming daughters (Fig. 5b arrows). We assessed the spatial relation of ATG8 relative to APP1 in detail by pan-ExM (Fig. 5c, Supplementary Movie 4). When daughter scaffolds are initiated (Fig. 5c, arrows), APP1 is directly located on the apicoplast outer membrane, with notable concentrations of ATG8 at the points where both proteins colocalize. Overall, ATG8 marks the apicoplast outer membrane and accumulates at the point where APP1 anchors the apicoplast in the early BC.

Interference with PI3P metabolism results in apicoplast inheritance defects^13–16^. Hence, we depleted ATG8 to determine whether APP1 remains associated with the DCS or with the apicoplast. Using streptavidin to visualize the apicoplast, we observed that APP1 localization remained largely unchanged in parasites lacking an apicoplast following ATG8-depletion. Specifically, a strong APP1 signal was detected at the apical end of the nucleus, where the centrosome resides, together with a weaker signal at the basal edge of the DCS (Fig. 5d). Hence, APP1 localization is not dependent on the apicoplast, highlighting the protein as a component of the DCS.

### APP1 proximity biotinylation identifies APP3

**Fig. 6:**
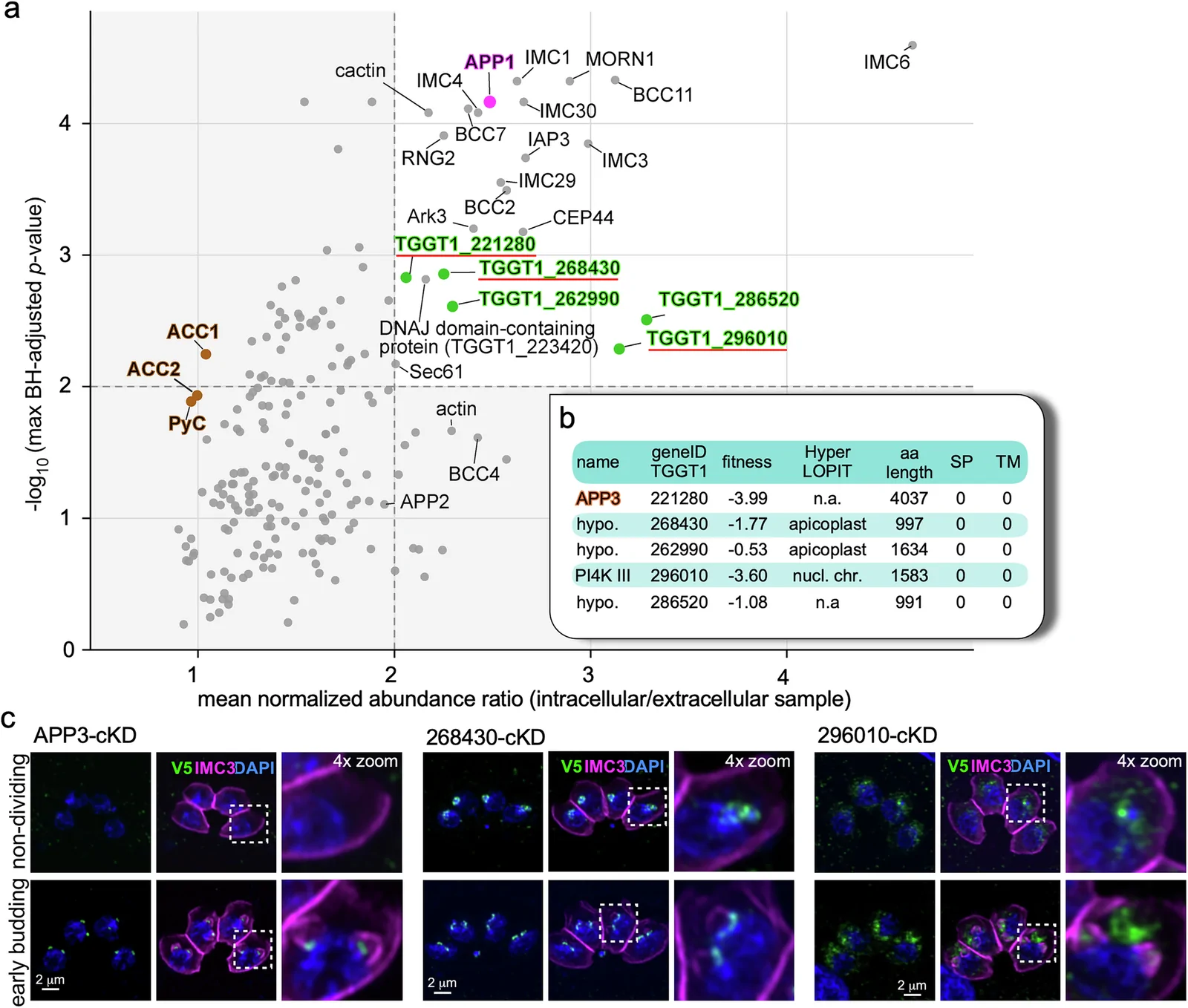
APP1 TurboID analysis. **a** APP1 TurboID candidates from two biological replicates. Brown-lined factors are endogenously biotinylated proteins; green-lined factors are hypothetical and unstudied proteins (red-underscored factors were selected for experimental validation). Note that APP2 (TGGT1_313360) was discovered and characterized in the co-submitted study by Mead et al.^30^ Mean normalized abundance ratios are plotted on the x-axis, and the y-axis displays -log_10_ of the maximum of the two replicate-specific Benjamini-Hochberg (BH)-adjusted *p*-values for each protein. **b** Table reporting on features of the identified candidates. Fitness data from ref. ^27^; HyperLOPIT organelle proteomics from ref. ^25^. **c** Localization of short-listed genes from **b**, colocalized with IMC3 in non-dividing and dividing parasites. Candidates were endogenously tagged with mAID and 5xV5 on either the 5’ or 3’ end of the ORF (Supplementary Fig 1e–g). DAPI stains DNA, Airyscan imaging of unexpanded samples.

To map putative interaction partners docking APP1 on the DCS or the apicoplast, we performed a TurboID experiment using APP1 as bait (Supplementary Fig. 1c, Supplementary Fig 5, 6). Given the dynamic localization of APP1 close to the plastid during division, we compared dividing parasites with non-dividing, extracellular parasites as a background biotinylation control. We focused on candidates that showed normalized enrichment ratios (intracellular sample/extracellular sample) > 2-fold as well as Benjamini-Hochberg adjusted *p*-values ≤ 0.01 in each biological replicate (Fig. 6a). The results comprised APP1 itself, validating the approach, as well as multiple BC proteins (BCC2, BCC7, BCC11, MORN1, and just at the cusp of significance, BCC4), multiple cytoskeleton scaffold proteins (IMC proteins 1, 3, 4, 6, 29, 30, RNG2) and centrosome-associated proteins (Ark3, CEP44, IAP3). In addition, we identified several annotated but unstudied proteins comprising a chaperone (DNAJ protein TGGT1_223420) and PI4K III (TGGT1_296010), as well as hypothetical proteins (TGGT1_286520, 262990, 268430 and 221280). Our dataset also included APP2 (TGGT1_313360), a constitutively expressed gene that was identified and characterized in the co-submitted study by Mead et al.^30^ (Fig. 6a).

We followed up on three genes (TGGT1_221280, TGGT1_268430 and TGGT1_296010), prioritized by significant fitness scores (<−2.5;^27^) or apicoplast localization by hyperLOPIT^25^ (Fig. 6b). For characterization, we inserted a mAID-5xV5 tag at the 3’-end of the endogenous ORFs. Using this strategy, we failed to obtain a discernible signal for TGGT1_268430 and TGGT1_296010 in indirect immunofluorescence assays using V5 antisera, prompting us to switch the tagging approach to one that targets the 5’-end of the ORF (Supplementary Fig. 1f, g). TGGT1_268430 exhibits a localization pattern reminiscent of the apicoplast (Fig. 6c), being in line with its previously assigned apicoplast localization by HyperLOPIT^25^. TGGT1_296010 has a more diffuse localization in non-dividing and dividing parasites (Fig. 6c). However, both parasite strains formed significantly smaller plaques (Supplementary Fig. 7), even in the permissive condition without auxin (IAA). This indicates that endogenous tagging alone already severely decreases parasite fitness in these strains, which led us to focus on TGGT1_221280.

### APP3 temporally resides in the centrosome and precedes APP1 appearance

**Fig. 7:**
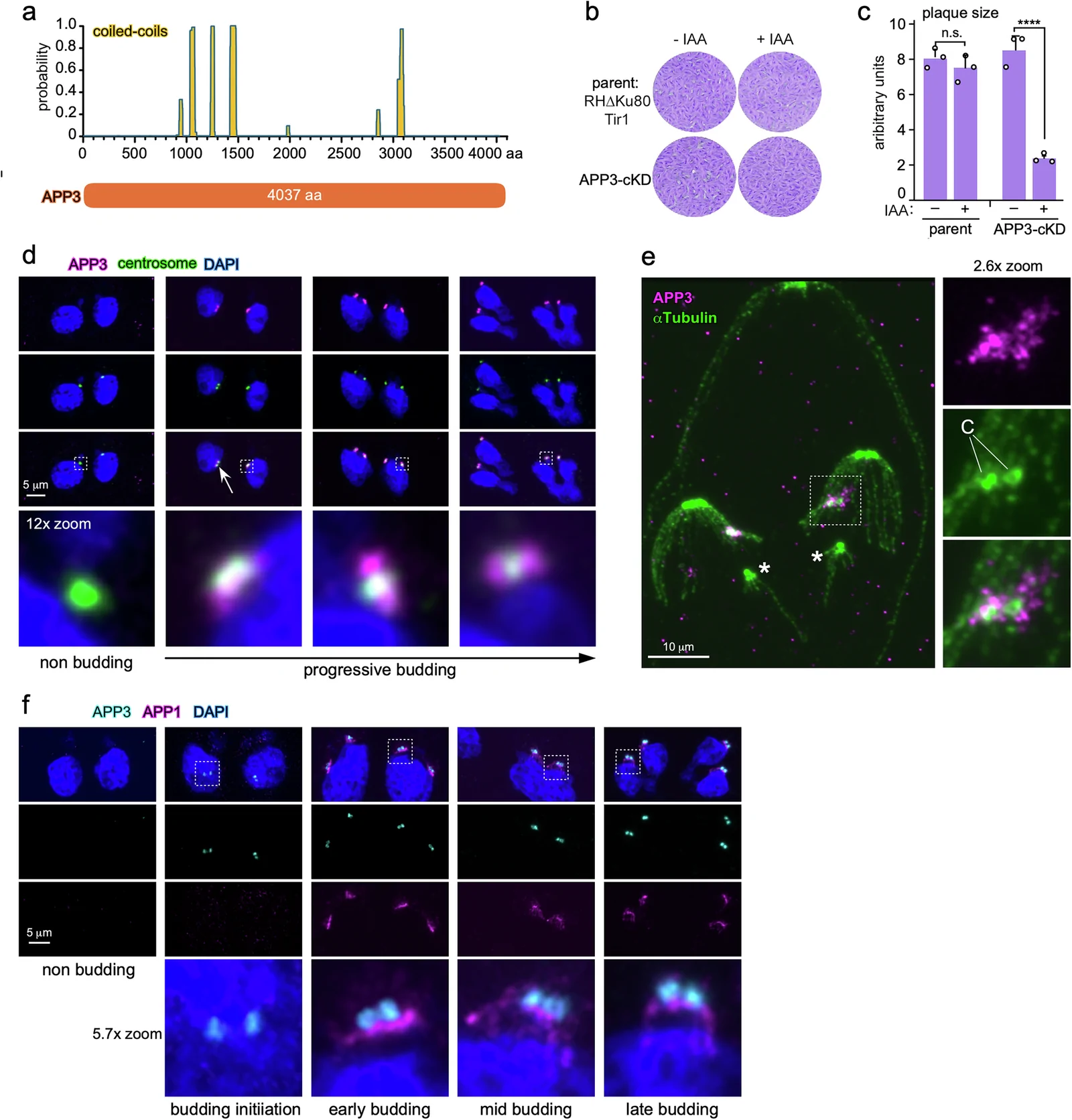
APP3 is critical for proliferation and localizes to the outer core of the centrosome prior to APP1. **a** Schematic of APP3 (TGGT1_221280), which is a 4037 aa protein, with five coiled-coil domains. For subsequent characterization, the gene was endogenously tagged at the 3’-end with a mAID and 5xV5 tag (APP3-cKD) in Tir1-ALFA-APP1 parasites. **b** Representative 7-day plaque assay showing that APP3-cKD in the presence of IAA forms small plaques, indicating a severe defect in completion of the lytic cycle. **c** Quantification of plaque size. a.u.=arbitrary units. Data represent mean values +std of *n* = 3 biological replicates. One-way ANOVA with Tukey HSD. NS= not significant, **** *p* = 1.13×10^−5^. **d** PS-ExM of non-dividing and dividing APP3-cKD tachyzoites, colocalized with the centrosome (Centrin (Cen20H5) antiserum). APP3 is not expressed in non-dividing parasites, but associates with the centrosome early in division, when the centrosome rotates to the basal side of the nucleus (white arrow). Throughout division, APP3 surrounds the centrosome. Images acquired via Airyscan imaging. Inlays are 12x zoom of boxed areas. DAPI = DNA. **e** Pan-ExM and Airyscan imaging of APP3-cKD verifies that APP3 localizes around the outer core of the centrosome (C: centrioles). Asterisks: spindle poles (inner core of the centrosome). Tubulin cytoskeleton visualized with α-Tubulin antiserum. **f** Time series of APP1 and APP3 colocalization, visualized by PS-ExM and Airyscan imaging. Both proteins are absent in non-dividing parasites, and APP3 precedes APP1 in localizing to the forming daughters (association with the outer core of the centrosome). Insets are 3x zoom of boxed areas. DAPI = DNA.

TGGT1_221280 is a 4037 amino acid protein with several coiled-coil regions as the only identifiable features (Fig. 7a). We named this candidate APP3. *T. gondii*’s cyst-forming relatives harbor an ortholog, but a candidate was not discernible outside this group. Depletion of APP3 resulted in significantly reduced plaque size (Fig. 7b, c), consistent with its severe fitness score of −3.99^27^. Subsequently, we tracked APP3 localization throughout cell division (Fig. 7d). APP3 is not expressed in non-dividing parasites, first appears when the centrosomes are duplicating, and is present on the lateral edges of the outer core of the centrosome throughout daughter budding (Fig. 7d; Centrin marks the centrosome outer core). We further detailed APP3 localization by pan-ExM co-stained with tubulin to mark the centrioles residing in the centrosome outer core. At the first appearance, recruitment stage, APP3 surrounds the centrioles, with the highest signal density closest to the centrioles (Fig. 7e). Hence, the consistently observed paired APP3 appearance is related to its centriole association.

To determine the relative timing of APP1 vs. APP3 recruitment to the forming daughter scaffolds as well as their relative localization, we visualized both proteins in the same parasite (Fig. 7f). We tracked parasites through the budding cycle, which showed that APP3 is present well before APP1 (Fig. 7f, bud initiation). Furthermore, it showed that the paired APP3 signal was located apical to APP1 signal associated with the BC (Fig. 7f, early budding) before APP1 stretches out along the DCS in association with the apicoplast. Especially the localization of APP3 on the very apical edge of APP1 on the apicoplast stands out and indicates the hierarchical assembly of the APP machinery.

### APP3 facilitates APP1 positioning on the BC and is essential for apicoplast partitioning

**Fig. 8:**
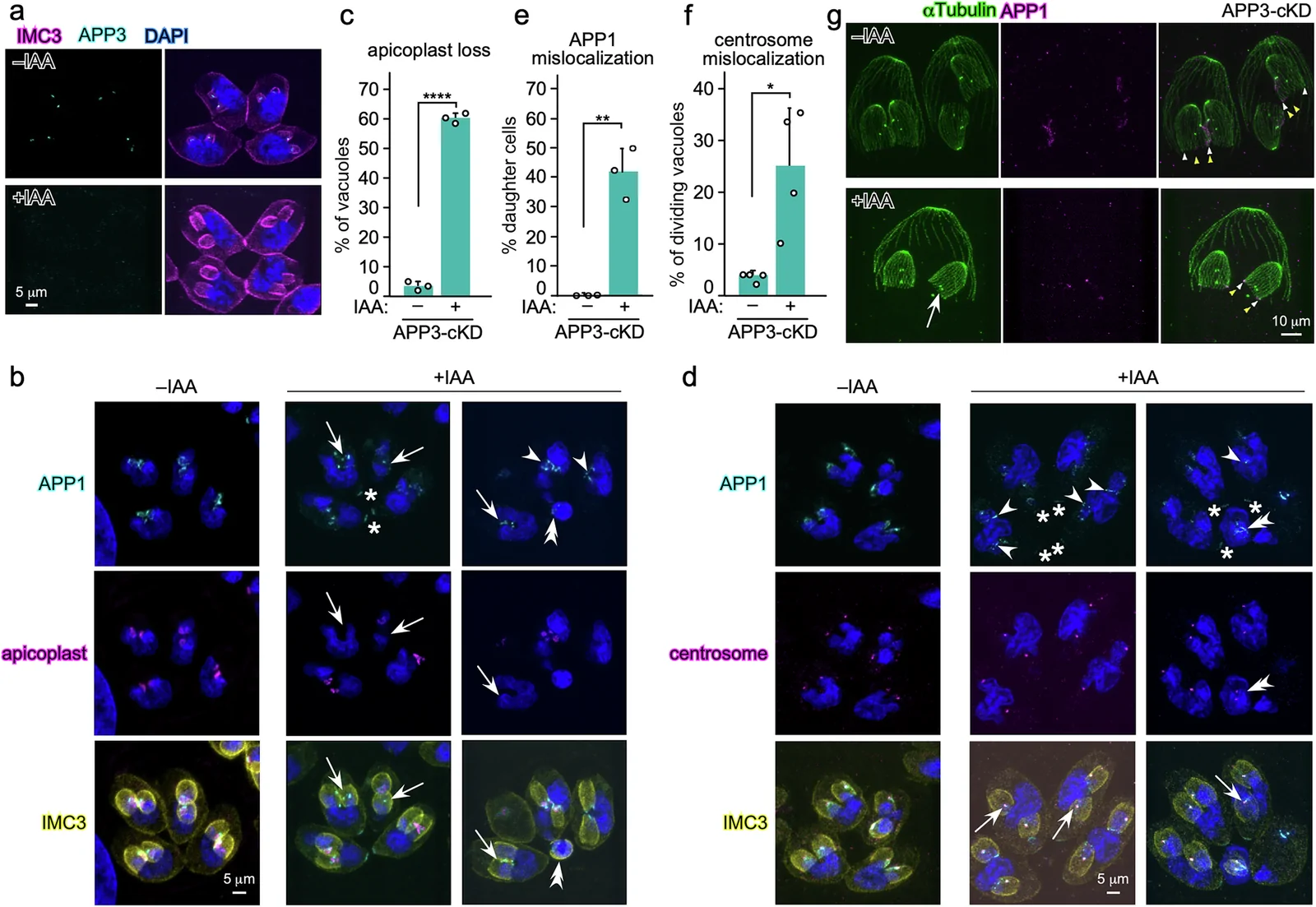
Depletion of APP3 affects apicoplast partitioning and localization of APP1. **a** The APP3-cKD strain loses APP3-V5 signal after treatment with IAA (24 hrs). PS-ExM with Airyscan imaging; DAPI stains DNA. **b** APP3 depletion leads to apicoplast partitioning defects. White arrows point to daughter cells where ALFA-APP1 is present without an apicoplast. Double arrowheads indicate ALFA-APP1, DNA. and IMC3 in the residual body. Arrowheads: increased ALFA-APP1 signal in the daughter BC, asterisk: ALFA-APP1 staining of the mature BC. PS-ExM with Airyscan imaging, DAPI stains DNA, apicoplast visualized via streptavidin. **c** Quantification of apicoplast partitioning defects in APP3-cKD parasites ± IAA treatment. Data represent mean values +std of *n* = 3 biological replicates with 100 vacuoles quantified per replicate. A two-tailed *t*-test was used to test for significance, **** *p* = 2.71×10^−7^. **d** Depletion of APP3 leads to mispositioning of the centrosome (highlighted via Cen20H5 antiserum, white arrows) and increased ALFA-APP1 localization to the daughter (arrowheads) and the mother (asterisk) BC. Double arrowheads indicate ALFA-APP1, centrosomes, DNA and IMC3 in the residual body. DAPI stains DNA. PS-ExM with Airyscan imaging. **e** Quantification of daughter cells that lost ALFA-APP1 localization to one side, with APP1 exhibiting daughter BC localization. PS-ExM with Airyscan imaging was used for quantification, ± IAA treatment. Data represent mean values +std of *n* = 3 biological replicates, with a total of 90 daughter cells quantified per condition. Two-tailed Welch’s *t*-test was used to determine significance, ** *p* = 0.0129. **f** Quantification of vacuoles with dividing parasites that exhibit centrosomal defects (e.g., mispositioning in daughter cells or centrosomes in the residual body), ±IAA treatment. Data represent mean values +std of *n* = 4 biological replicates, with 50 vacuoles harboring dividing parasites quantified. Two-tailed Welch’s *t*-test used to determine significance, * *p* = 0.0397. **g** APP3 confines APP1 to one side of the forming daughter. Pan-ExM of APP3-cKD parasites, − IAA or + IAA treatment for 24 hrs. Tubulin structures are visualized via α-Tubulin (12G10) staining, APP1 is visualized with ALFA antiserum. The widths of the daughter cell basal ends are highlighted by white arrowheads, and the width of APP1 signal at the daughter basal end by yellow arrowheads. White arrows indicate misplaced centrioles in daughter cells. Images were acquired via Airyscan microscopy.

We interrogated APP3 function by depleting the protein using the mAID system, which showed APP3 was undetectable in parasites grown for 24 hrs in the presence of IAA (Fig. 8a). Furthermore, APP3-depleted parasites showed a severe apicoplast partitioning defect, with 60% (±1 std) of vacuoles harboring at least one parasite with a missing organelle (Fig. 8b, c). We further noticed that APP1, while present in daughter buds in the absence of the apicoplast (Fig. 8b, white arrows), was frequently mislocalized in APP3-depleted parasites. Here, APP1 was detected at the daughter BC (Fig. 8b, d, arrowheads) in 42.06% (± 8.36 std) of daughter cells (Fig. 8e) and failed to localize to one side of the forming buds. APP1 further exhibited localization to the mother BC (Fig. 8b, d, asterisks). Additionally, in 25% (±12.27 std) of vacuoles harboring dividing parasites, we observed compromised centrosomes (Fig. 8f). These centrosomes were mispositioned and remained close to the daughter BCs, thereby failing to move into the upper one-third of the forming daughter buds (Fig. 8d, white arrows). In the most dramatic cases, centrosomes together with APP1, nuclear and IMC3 material ended up in the residual body (Fig. 8b, d, double arrowheads).

We corroborated the above-identified phenotypes at higher resolution by pan-ExM (Fig. 8g). This confirmed that APP1 is confined to one side of the daughter bud in control parasites (Fig. 8g, -IAA yellow arrowheads), while localizing to the entire daughter BC in the absence of APP3 (Fig. 8g, +IAA yellow arrowheads). Pan-ExM also showed that the outer core of the centrosome (highlighted via tubulin staining of the centrioles), was closer to the daughter BC (Fig. 8g, +IAA arrow) then seen in controls.

Taken together, APP3 depletion results in several cell division defects. Regarding apicoplast inheritance, APP3 depletion leads to APP1 mislocalization, which suggests that APP3 acts upstream of APP1 to promote its positioning on the subregion of the BC in close apposition to the apicoplast.

## Discussion

In this study, we identified several factors with anchoring roles crucial for directed inheritance of the apicoplast. Although a role for the centrosome was recognized before^9^, the molecular machinery connecting the centrosome and the apicoplast had remained elusive. Here, we characterized two proteins that are critical for the centrosome-apicoplast connection in *T. gondii*, which further revealed that a special section of the DCS (between the centrosome and BC) as well as the BC itself are also involved in the partitioning process. The centrosome and BC serve as anchoring points and position cues for APP proteins, which connect the apicoplast to the cell division machinery. Indeed, this is consistent with the previously observed involvement of the BC in apicoplast partitioning^22,23^ and is further reinforced by APP1 being the top hit in the BCC4 proximity biotinylation experiment (Fig. 1a). The BC and DCS are unique apicomplexan structures and therefore emphasize the directed evolutionary nature of the apicoplast partitioning machinery. We note that the partitioning infrastructure is only expressed during the cell division process, explaining the ‘ameboid’-like dynamics of the apicoplast in G1, before the organelle takes on a rigid appearance through centrosomes and DCS anchoring^31,32^. In a mirrored principle, the *T. gondii* mitochondrion is also anchored to the DCS (but not the centrosome or BC)^7^, thereby highlighting a crucial role for the cortical membrane cytoskeleton in single copy organelle partitioning.

The APP proteins do not contain any functional domains and are composed of low complexity regions, harboring some coiled-coil domains as their only recognizable feature. Such structural motifs are typically present in fast-evolving proteins of which centrosome-associated proteins are a prime example^33^. APP3’s centriole apposition and primary structure are consistent with this centrosome-protein-derived evolutionary APP model. Moreover, this appears to be a common feature of proteins in DCS-related structures, as the apical annuli—embedded in the IMC sutures—are also composed of similar, likely centrosome-derived proteins^34^ (we note that apical annuli protein 1 (AAP1) was initially short-listed as a CEP250-related protein, due to its high similarity score^11^). The fast-evolving nature of these proteins is reflected in their conservation pattern across Apicomplexa, with clear APP orthologs only identifiable in the close Coccidian relatives of *T. gondii*. Based on this, we cannot exclude the presence of orthologous APPs outside Coccidia. However, other factors could be uniquely tailoring the organelle partitioning machinery to the distinguishing features of the respective cell division strategy and/or architectural differences of the DCS seen in different Apicomplexa^35^. For example, during schizogony in *Plasmodium* parasites, the apicoplast and mitochondrion are tethered together in the cell division process^36–39^. In contrast, during gametocytogenesis the organelles are inherited independently in both species as the female gamete retains the apicoplast and the male gamete carries the mitochondrion^40,41^. Detailing the shared and different molecular mechanisms across these different scenarios and principles will be an exciting avenue for future work.

Besides revealing the general principles of the apicoplast partitioning machinery, we can assign specific functions to different APP proteins. APP3 is the first protein to appear right when the centrosomes duplicate, and it resides around the centrioles in the centrosome’s outer core. Subsequently, APP1 is recruited upon BC formation, where it is deposited in a defined sub-region (without APP3, APP1 is present on the entire BC). This BC sub-region is on the facing edges of the assembling DCS, which is the same side on which the centrosomes are anchored in the DCSs (Supplementary Fig 2, Supplementary Movie 1). When budding progresses, APP1, together with the apicoplast, is present along the DCS region between the centrosome and BC. Although apicoplast anchoring to the centrosome appears to be the most critical, our data indicate that association with the DCS and BC also contributes to apicoplast anchoring. Furthermore, the apicoplast fission machinery, notably dynamic related protein A (DrpA), is positioned at the basal ends of the DCS, where the BCs of neighboring buds meet^42^. How critical the exact positioning of DrpA is, is currently not fully understood, as apicoplast fission can occur in the absence of budding (e.g., upon disruption of microtubules via oryzalin^31^). The observation of multiple streptavidin foci corresponding with the number of division cycles summed across parasites in the same vacuole supports that loss of DCS anchoring does not prevent apicoplast fission (Fig. 4a). However, additional work is needed to resolve the details on a putative functional connection with fission.

Although our work reveals how the APP machinery is anchored to the DCS, it is not completely clear how it is connected to the apicoplast. We note an enrichment of apicoplast outer-membrane-residing ATG8 exactly at the sites where APP1 is observed (Fig. 5c and Supplementary Movie 4), suggesting a potential functional association. Consistent with this model, depletion of ATG8 resulted in apicoplast loss while APP1 remained on the DCS (Fig. 5d). On the other hand, APP1 TurboID did not detect ATG8, indicating that the two proteins likely do not directly interact. However, it is also possible that the membrane-embedded nature of ATG8 prevents efficient biotinylation. Another possibility is that the putative Apolipophorin-III (apo-LpIII) domain in APP1 is involved in apicoplast binding. In vertebrates, Apolipoproteins L1 and L3 are Golgi-associated with the membrane fission factor phosphatidylinositol 4-kinase-IIIβ (PI4Kβ)^43^. Furthermore, APOL3, along with APOL1, plays a role in controlling mitochondrial membrane dynamics through interactions with other proteins like PI4Kβ and VAMP8^43^. In this light, TgPI4KIIIβ was detected in both the BCC4 and APP1 proximity biotinylation experiments, but its severe fitness decrease even in the absence IAA led us to not explore it further in this study. Either way, elucidating the potentially multi-factorial nature of the connection to the apicoplast will require further work in the future.

Recently, PfAnchor was described in *P. falciparum*, which localizes to the apicoplast and is required for recruiting the fission-driving, dynamin-like GTPase PfDyn2^44^. Since PfAnchor orthologs were not found outside *Plasmodium* species, the localization of the apicoplast fission machinery appears to be executed by different, so far unknown, components in *T. gondii*. However, a functionally orthologous mechanism is anticipated.

In conclusion, the APP family, identified by Mead et al.^30^ and us, fills in a long-standing knowledge gap in apicoplast biology. Both studies assign a clear role to the centrosome outer core in staging the machinery for apicoplast partitioning, but highlight previously unappreciated supportive roles for the DCS and BC in executing the process. The centrosome deposition and centrosome-derived nature of APP proteins further expands the pivotal function of the centrosome in coordinating the events in apicomplexan cell division^11^.

## Methods

### Parasites and mammalian cell lines

Transgenic derivatives of the *T. gondii* RH strain were maintained in hTERT-immortalized human foreskin fibroblasts (hTERT-HFFs) as previously described^45^. The RH-TaTiΔKu80ΔHXGPRT strain was kindly shared by Dr. Lilach Sheiner, University of Glasgow. Parasite transgenic selections were performed under 1 μM pyrimethamine, 20 μM chloramphenicol, or a combination of 25 mg/ml mycophenolic acid and 50 mg/ml xanthine (MPA/X).

### Plasmids and parasite strain generation

All oligonucleotides used in this study are listed in Supplementary Table 1. Endogenous tagging was performed using a CRISPR/Cas9-based strategy with subsequent homology-directed repair to integrate desired modifications. Protospacer sequences targeting Cas9 to respective localizations in the *T. gondii* genome were cloned via Gibson Assembly into the pU6-Universal plasmid (kindly provided by Sebastian Lourido, Whitehead Institute, MA (Addgene #52694)^46^. For promoter replacement of APP1, a CRISPR/Cas9 construct was designed to create a DNA double-strand break close to the start ATG. For homology-directed repair, a PCR amplicon was cotransfected, which consists out of the DHFRTS resistant marker cassette, the TetO7sag4 regulatable promoter, and the Ty-1 epitope tag^24^, flanked by short 35 bp homology arms on each side. For endogenous 5’-tagging of APP1 with either the ALFA tag or a Ty-TurboID construct, the same CRISPR/Cas9 plasmid was used, but the PCR amplicon for homology-directed repair consisted of the HXGPRT ORF, the T2A skip peptide sequence, the ALFA tag^47^ or Ty-TurboID, again flanked by 35 bp homology arms. A similar strategy was used to 5’-tag BCC4 (TGGT1_229260) with Ty-TurboID. HX-T2A-ALFA construct was also transfected into RH∆Ku80-Tir1 parasites^48^, resulting in the Tir1 ALFA-APP1 line.

The Tir1 ALFA-APP1 line was used to endogenously tag ATG8 (TGGT1_254,120), TGGT1_268,430, or PI4KIII (TGGT1_296010) at the 5’-end of the ORF, while APP3 (TGGT1_221280) was tagged at the 3’end of the ORF with a mAID-5xV5 construct. Clonal lines were established via limiting dilution, and correct integration was verified by diagnostic PCR using specific oligonucleotide sets (Supplementary Fig. 1 and Supplementary Table 1).

### Plaque assays

Plaque assays were performed with confluent monolayers of hTERT-HFFs. Freshly lysed parasites were filtered through a 3 μm filter, inoculated onto host cell monolayers ± 500 μM IAA, or ± 0.5 mg/ml, ATc and allowed to form plaques for 7–9 days. Cell layers were fixed with 100% ethanol, stained with crystal violet, and washed 3 times in 1xPBS^45^. Plaque size was quantified using FIJI^49^.

### (Immuno-) fluorescence microscopy

Indirect immunofluorescence assays were performed on intracellular parasites grown for 24 hrs in 6-well plates ± 500 μM IAA, or ± 0.5 mg/ml ATc, containing coverslips confluent with HFF cells. Samples were fixed with 100% methanol or 4% paraformaldehyde (PFA) in 1xPBS followed by permeabilization with 0.25% TX-100 in 1xPBS. Primary antisera used are listed in Supplementary Table 2. Streptavidin, conjugated to either Alexa488 (A488) or Alexa594 (A594), was used to visualize the apicoplast (1:1000; ThermoFisher). A488, A594A647-conjugatedated goat α-mouse, α-rabbit, α-rat, or α-guinea pig were used as secondary antibodies (1:500, ThermoFisher). DNA was stained with 4’,6-diamidino-2-phenylindole (DAPI).

Images were acquired on either a DeltaVision wide-field microscope, Zeiss AxioImager Z2 with Apotome, or Zeiss LSM 880 confocal microscope with Airyscan unit. Images acquired with Zeiss microscopes were captured with Zeiss ZenBlue or Black and deconvolved using ZenBlue with standard settings. Final image analyses/adjustment of brightness and contrast were made with FIJI software. For phenotype quantifications, slides were blinded to the experimenter, and 50–100 vacuoles at random fields were counted. All antibodies used in this study can be found in Supplementary Table 2.

### Iterative ultra-structure expansion microscopy (pan-ExM)

Pan-ExM was carried out as we previously reported^50^. Briefly, tachyzoites were grown overnight in 6-well dishes on confluent HFF cells, treated for indicated time frames with ATc or IAA and fixed with 4% PFA. Samples were incubated in anchoring solution (0.7% formaldehyde, 1% acrylamide in PBS) for 3 hrs at 37 °C. To achieve a consistent primary gel thickness, samples were placed in gelation chambers^51,52^ and incubated with primary gel solution (19% sodium acrylate, 10% acrylamide, 0.1% N,N’-(1,2-dihydrooxyethylene)bisacrylamide (DHEBA) in PBS, 0.25% TEMED and APS) for 1 hr at 37 °C. Gels were first denatured in a buffer (200 mM SDS, 200 mM NaCl, 50 mM Tris, pH 6.8) for 15 min at RT and subsequently incubated at 73 °C for 1 hr, and then left for overnight expansion in ultrapure water. A 2 × 2 cm gel piece was taken for further processing and incubated in neutral hydrogel solution (10% acrylamide, 0.05% DHEBA, in H_2_O, 0.05% TEMED and APS), before gels were sandwiched between a microscopy slide and a 22 mm coverslip, followed by an 1 hr incubation at 37 °C. Gels were then incubated in secondary gelation solution (19% sodium acrylate, 10% acrylamide, 0.1% bis-acrylamide in PBS, 0.05% TEMED and APS), again sandwiched between a microscopy slide and a 22 mm coverslip, and incubated for 1 hr at 37 °C. To dissolve DHEBA, gels were placed in 0.2 M NaOH in H_2_O for 1 hr at RT and subsequently washed in PBS until the pH was neutral. Pieces of the final gel were used for primary antibody staining with antibodies listed in Supplementary Table 2 overnight at RT, followed by a 3 hrs incubation step at 37 °C at the next day. Gels were washed in 0.1% PBST before being incubated in secondary antibody solution with the same incubation times and temperature conditions used for primary antibodies. Gels were washed in 0.1% PBST before the final expansion was done in ultrapure water. Expanded gels were then placed in 35 mm glass bottom dishes (Ibidi) coated with poly-L Lysine and imaged on a ZeissLSM880 microscope with Airyscan unit. Images were acquired with Zeiss Zen Black software, while Zeiss Zen Blue version 2.3 was used for image processing using standard settings. Final image processing was done in FIJI by adjusting brightness and contrast.

### Pre-gelation staining expansion microscopy (PS-ExM)

PS-ExM samples were generated as previously reported^26^. In short, tachyzoites growing in HFF cells for 24 hrs ± ATc or IAA were fixed with 4% PFA and permeabilized with 0.25% TX-100 in PBS for 10 min. IFA staining was performed as stated above, but DAPI staining was omitted. Coverslips were then incubated in pan-ExM anchoring solution for 90 min at 37 °C and gelation was done in pan-ExM primary gel solution for 30 min at 37 °C. Gels were incubated in pan-ExM denaturation buffer at 73 °C for 45 min, briefly washed in 1xPBS, and incubated in DAPI solution (1 μg/ml in PBS) for 30 min. Finally, gels were incubated in ddH_2_O for expansion overnight, mounted in poly L-lysine coated 35 mm glass bottom microwell dishes (Ibidi) the next day, and imaged on a Zeiss LSM880 with Airyscan unit using standard settings for image acquisition and Airyscan deconvolution. Image processing was done in FIJI to generate 2D projections that were adjusted for brightness and contrast.

### qPCR of the apicoplast genome

TATi∆HXGPRT and APP1-cKD parasites were grown with or without ATc for 72 hrs before total DNA was extracted with the DNA Blood&Tissue kit (Qiagen). The Power SYBR Green PCR master mix (Thermo Fisher) together with 50 ng of parasite DNA and 0.35 μM of oligonucleotides that either amplify actin (encoded in the nuclear genome) and TogoCR29 (encoded in the apicoplast genome) (Supplementary Table 1), was used in 10 μl qPCR reactions, as previously reported^53,54^. Each oligonucleotide/DNA combination was run in triplicate for each of the three biological replicates executed. All qPCR reactions were performed using 60°C for oligonucleotide annealing and 72°C for product elongation using an Applied Biosystems QuantStudio3 Real-Time PCR System. A dissociation step was performed after each individual qPCR run. To determine differences in gene copy number, the 2^-∆∆Ct^ method was used as previously reported^53,55^, indicating the fold change of the apicoplast genome compared to the nuclear genome.

### Proximity biotinylation and mass spectrometry

Proximity biotinylation was done as previously reported^24^. For each TurboID data set, two biological replicates that were split into two technical replicates respectively, were done. To specifically enrich for division-related proteins, a low parasite inoculum ( ~ 1.2*10^6^ per T175) was used and cultured for 30 hrs with a subsequent 2 hrs biotin application (500 μM). As a control, the same parasite line was used in a high inoculum ( ~ 4*10^6^), grown identically, to allow complete lysis of the host cell layer. Control parasites were then incubated for an additional 4 hrs to allow all parasites to complete cell division before a 2 hrs incubation with 500 μM biotin was started. Host cells were mechanically lysed in case of the division sample and all samples were filtered through 3 μm nylon membranes and washed several times in ice-cold PBS before parasite lysis was achieved with 1% SDS in resuspension buffer (150 mM NaCl_2_, 50 mM Tris-HCl, pH 7.4, protease inhibitor (Roche)). Lysates were boiled and cleared by centrifugation before adjusted to 1.2% SDS in DPBS (Corning) and incubated with streptavidin-agarose beads (Fisher) overnight at 4°C. Beads were washed with 0.1% SDS in DPBS, DPBS and H_2_O the next day, resuspended in 6 M Urea, reduced, alkylated and digested with 2 μg trypsin overnight at 37°C. Digested peptides were separated from beads by centrifugation, and either directly used for mass spectrometric analyses (BCC4 TurboID) or subsequently labeled with Tandem Mass Tags (TMT) (APP1 TurboID).

For BCC4 samples, Liquid chromatography-tandem mass spectrometry (LC-MS/MS) was performed on an Orbitrap Exploris 240 mass spectrometer with Xcalibur version 4.4 (Thermo Fisher Scientific) coupled to a Dionex UltiMate 3000 RSLCnano system as previously reported^24^. LC-MS/MS data were analyzed with Thermo Proteome Discoverer version 2.4 and searched using SequestHT and Percolator algorithms against *Homo sapiens* proteome database and the *T. gondii* proteome sourced from VEuPathDB (ToxoDB, GT1 annotation version 48;^56^). The protease for data analysis was specified as trypsin with a maximum of 2 missed cleavages. Peptide precursor tolerance was set to 10 ppm with a fragment mass tolerance of 0.02 ppm. Cysteine alkylation ( + 57.021) was set as a static modification, methionine oxidation ( + 15.995), acetylation ( + 42.011), and methionine loss ( + 131.040) of the protein N-terminus were set as dynamic modifications. The false discovery rate (FDR) was set to 1% for peptide identification.

For TMT-labeled APP1 samples, peptides were incubated at RT with 60 μg of TMT reagent (Thermo Fisher) for 1 hr, before the reaction was stopped with hydroxyamine. Samples were acidified, combined, and desalted with a SEP PAK C18 cartridge and subjected to mass spectrometry. The samples were run on an Orbitrap Exploris 240 mass spectrometer with Xcalibur version 4.4 and analyzed using Thermo Proteome Discoverer V2.4. TMT 6plex (+229.163) modification on lysine residues and protein N-terminus was set as static modifications in addition to cysteine alkylation by IA ( + 57.021). For TMT-based quantification, a centroid tolerance of 20 ppm was applied for integration, with a co-isolation threshold of 50% and an average reporter signal-to-noise threshold of 10. *p*-values of individual protein abundances were calculated by one-way ANOVA and adjusted using the Benjamini-Hochberg procedure. The Proteome Discoverer protein output tables for the two experiments are provided in Supplementary Data 1.

Protein abundance ratios were normalized within each replicate to the mean abundance ratio of endogenously biotinylated ACC1 (TGGT1_221320), ACC2 (TGGGT1_2185660), and pyruvate carboxylase (TGGT1_284190).

### Ortholog mapping and phylogeny

APP proteins were BLASTP searched against VEUPathDB against the following representative reference genomes: BbesnoitiBbGer1, BbigeminaBOND, Bdivergens1802A, BduncaniWA12023, BmicrotiRI, CcayetanensisCHN_HEN01, CfelisWinnie, CparvumIowaII, CsuisWienI, CveliaCCMP2878, EacervulinaHoughton, EmaximaWeybridge, EtenellaHoughton2021, GniphandrodesUnknown, HhammondiHH34, NcaninumLIV, PbergheiANKA, Pfalciparum3D7, PgiganteaA, PknowlesiH, PvivaxP01, Pyoeliiyoelii17X, SneuronaSN3, TannulataAnkara, TgondiiME49, VbrassicaformisCCMP3155. Protein sequences were aligned and the phylogram was generated by Clustal Omega 1.2.4^57^. The Percent Identity Matrix was calculated by Clustal2.1.

### Western blotting

TurboID-APP1 or parental parasites were grown for 30 hrs under standard culture conditions, then + biotin samples were treated with 500 μM biotin for 2 hrs. Parasites were released from host cells via needle lysis, filtered through a 3 μm nylon filter membrane and lysed in 150 mM NaCl, 50 mM Tris-HCl, pH 7.4, Protease inhibitor and 1% SDS. For western blotting, lysates of 2 × 10^6^ parasites per lane were separated via SDS-PAGE and proteins were transferred on PVDF membranes. Membranes were blocked in 6% milk in PBS and subsequently stained with streptavidin-HRP in 5% BSA in PBS. Super Signal West Atto Ultimate Sensitivity Substrate (ThermoFisher) was used as the HRP substrate.

### Statistics and reproducibility

Statistical analyses were performed in R and Microsoft Excel. For comparisons between two conditions, statistical significance was assessed using two-tailed Welch’s *t*-test, whereas one-way ANOVA followed by Tukey’s HSD post hoc test was used for comparisons involving three or more conditions. *P*-values < 0.05 were considered statistically significant. Unless otherwise stated, three biological replicates (*n* = 3) were used for the experiments, and data presented as mean + std. All microscopy images are representative of at least two independent experiments. All experiments yielded in comparable results. For the APP1 TurboID experiments, the mean of the two normalized protein abundance ratios was used as the fold-enrichment measure. Replicate consistency was summarized using the maximum of the two replicate-specific Benjamini-Hochberg-adjusted *p*-values, with each replicate evaluated independently rather than calculating a combined *p*-value.

### Reporting summary

Further information on research design is available in the Nature Portfolio Reporting Summary linked to this article.

## Supplementary information

Supplementary Information

Supplementary Data 1

Supplementary Movie 1

Supplementary Movie 2

Supplementary Movie 3

Supplementary Movie 4

Reporting Summary

Transparent Peer Review file

## Data Availability

The mass spectrometry proteomics data have been deposited to the ProteomeXchange Consortium via the Pride^58^ partner repository with the dataset identifier PXD075456. *T. gondii* genome information can be found in ToxoDB^59^ (https://toxodb.org). Source data are provided with this paper.
