# Supplementary material for: A dedicated partitioning machinery ensures faithful inheritance of the apicoplast in Toxoplasma gondii
Source: Nat Commun. 2026 Oct 5;17:10368. doi: 10.1038/s41467-026-78103-w (PMC13639030; doi:10.1038/s41467-026-78103-w)
Supplement: Supplementary file 1 [file 41467_2026_78103_MOESM1_ESM.pdf]

# Supplementary Information

## A dedicated partitioning machinery ensures faithful inheritance of the apicoplast in *Toxoplasma gondii*

Garret C. Eichlin[1§], Prince Oppong[1§], Hallie R. Hayne[1], Pamodya Pamunuwa[2], Benjamin Chartier[2], Eranthie Weerapana[2], Marc-Jan Gubbels[1], Klemens Engelberg[1]*

[1] Department of Biology, Boston College, Chestnut Hill, MA, USA
[2] Department of Chemistry, Boston College, Chestnut Hill, MA, USA

[§] These authors contributed equally
*Corresponding author: engelbek@bc.edu

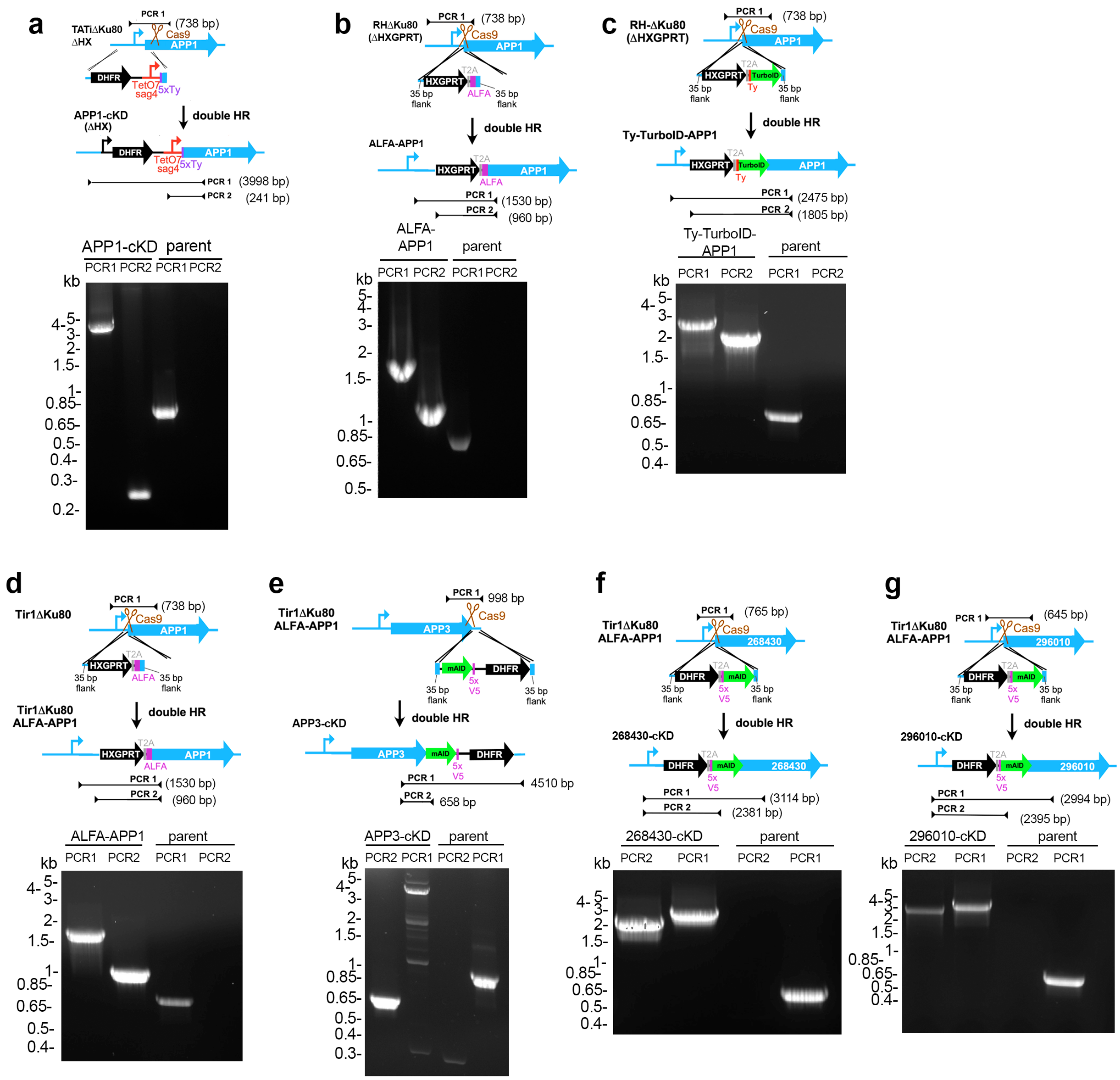


**Supplementary Figure 1. Diagnostic PCRs of the established transgenic parasite lines.** Schematics indicate the modification in the respective gene locus. The calculated product sizes for each PCR are indicated in the schematics. HR: homologous recombination, kb: kilo base pairs. All oligonucleotides used are listed in **Supplementary Table 1**. a: APP1-cKD; b: ALFA-APP1 in RHΔKu80; c: TurboID-APP1; d: ALFA-APP1 in Tir1ΔKu80; e: APP3-cKD; f: 268430-cKD and g: 296010-cKD.

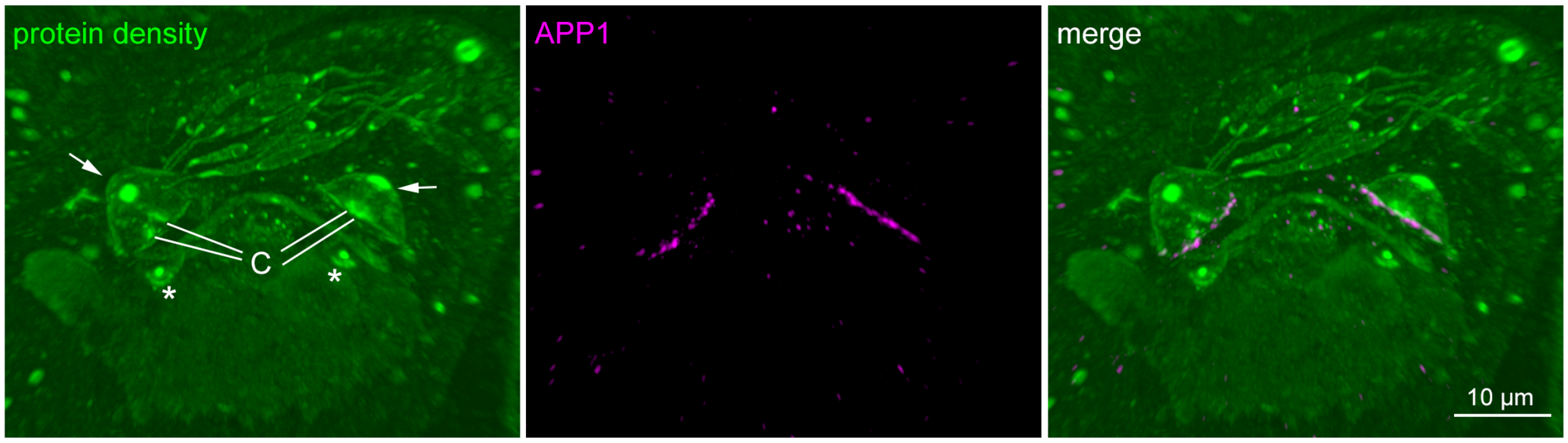


**Supplementary Figure 2. APP1 localizes to only one side of the daughter BC, early in the budding.** Pan-ExM with subsequent Airyscan imaging. Total protein was stained with NHS-ester-ATTO488, APP1 was visualized via ALFA antiserum. C: centrioles; asterisks: spindle poles; arrows: DCS. For 3D rotation see **Supplementary Movie 1**.

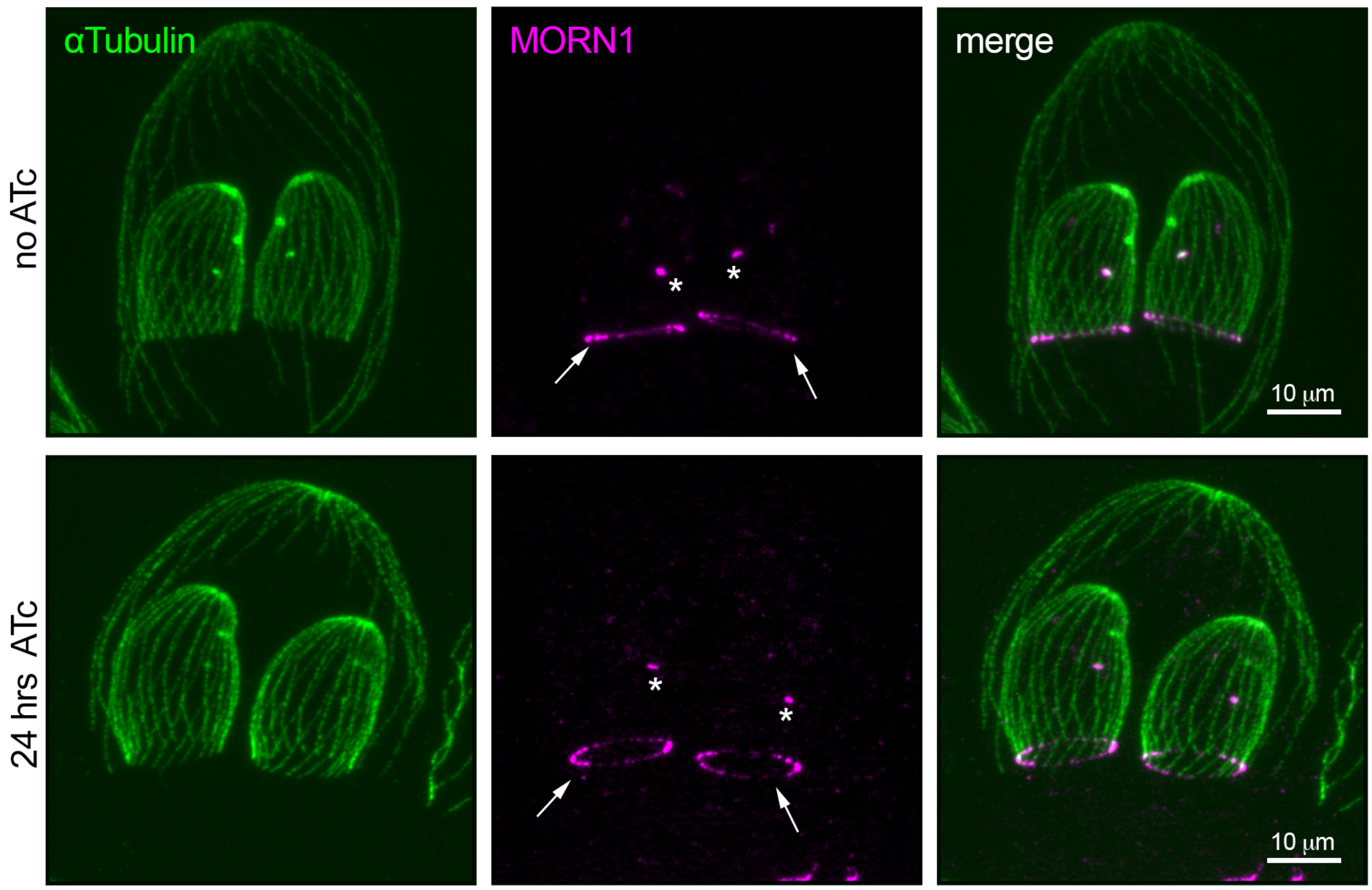


**Supplementary Figure 3. APP1 knockdown does not induce morphological changes to the parasite.** APP1-cKD parasites, treated or not treated with ATc for 24 hrs were subjected to pan-ExM with subsequent Airyscan imaging. Samples were stained with αTubulin and MORN1 antiserum, to highlight the tubulin cytoskeleton and MORN1 structures, respectively. The MORN1 centrocone signal is highlighted with asterisks, MORN1 signal at the daughter BCs with arrows.

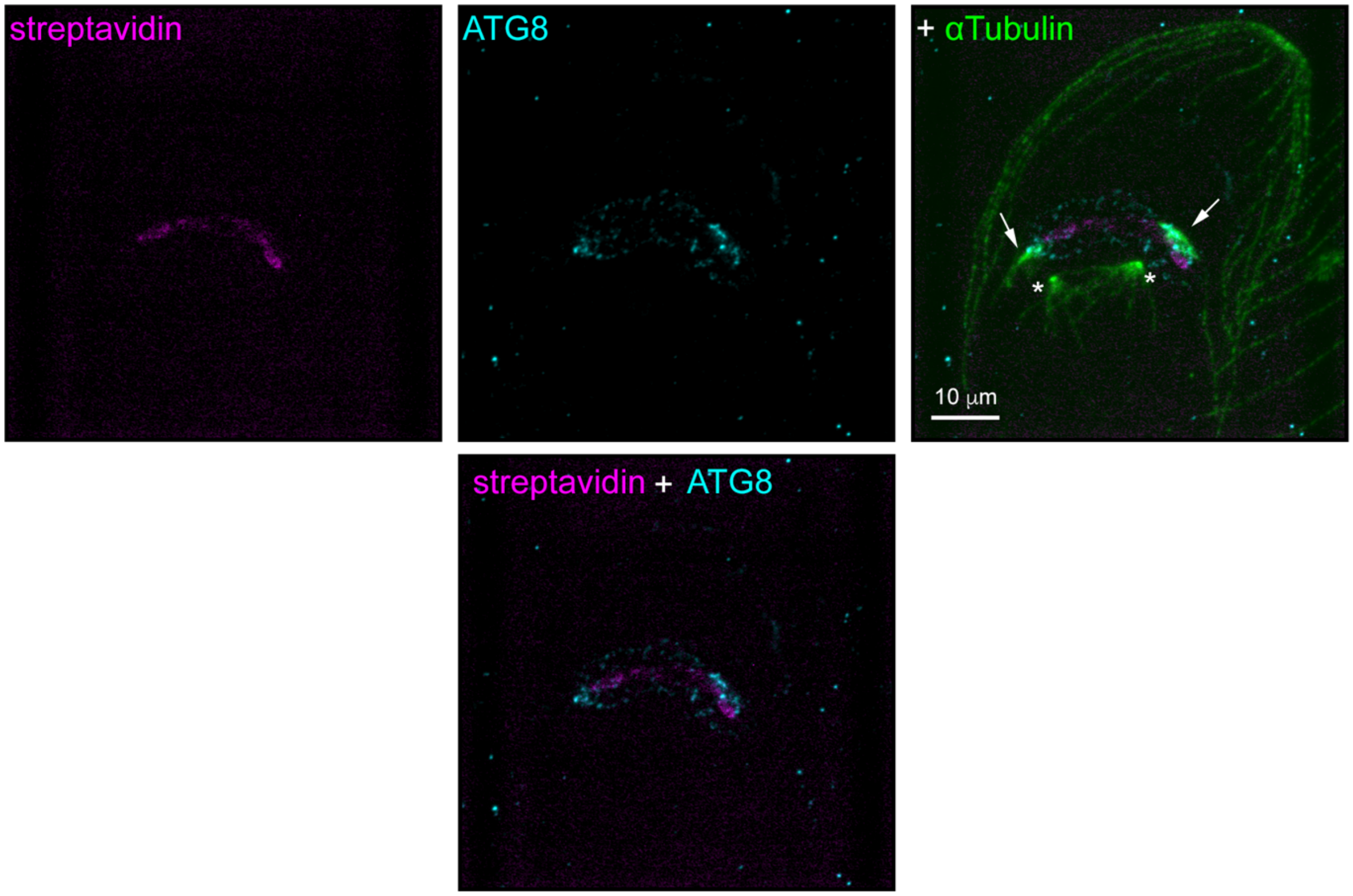


**Supplementary Figure 4. ATG8 highlights the apicoplast membrane, surrounding the luminal streptavidin-staining.** Pan-ExM with subsequent Airyscan imaging. The apicoplast lumen is visualized via streptavidin-A594, V5-mAID-ATG8 is visualized with V5 antiserum. Forming daughter buds are highlighted by arrows, spindle poles by asterisks. For 3D rotation see **Supplementary Movie 3**.

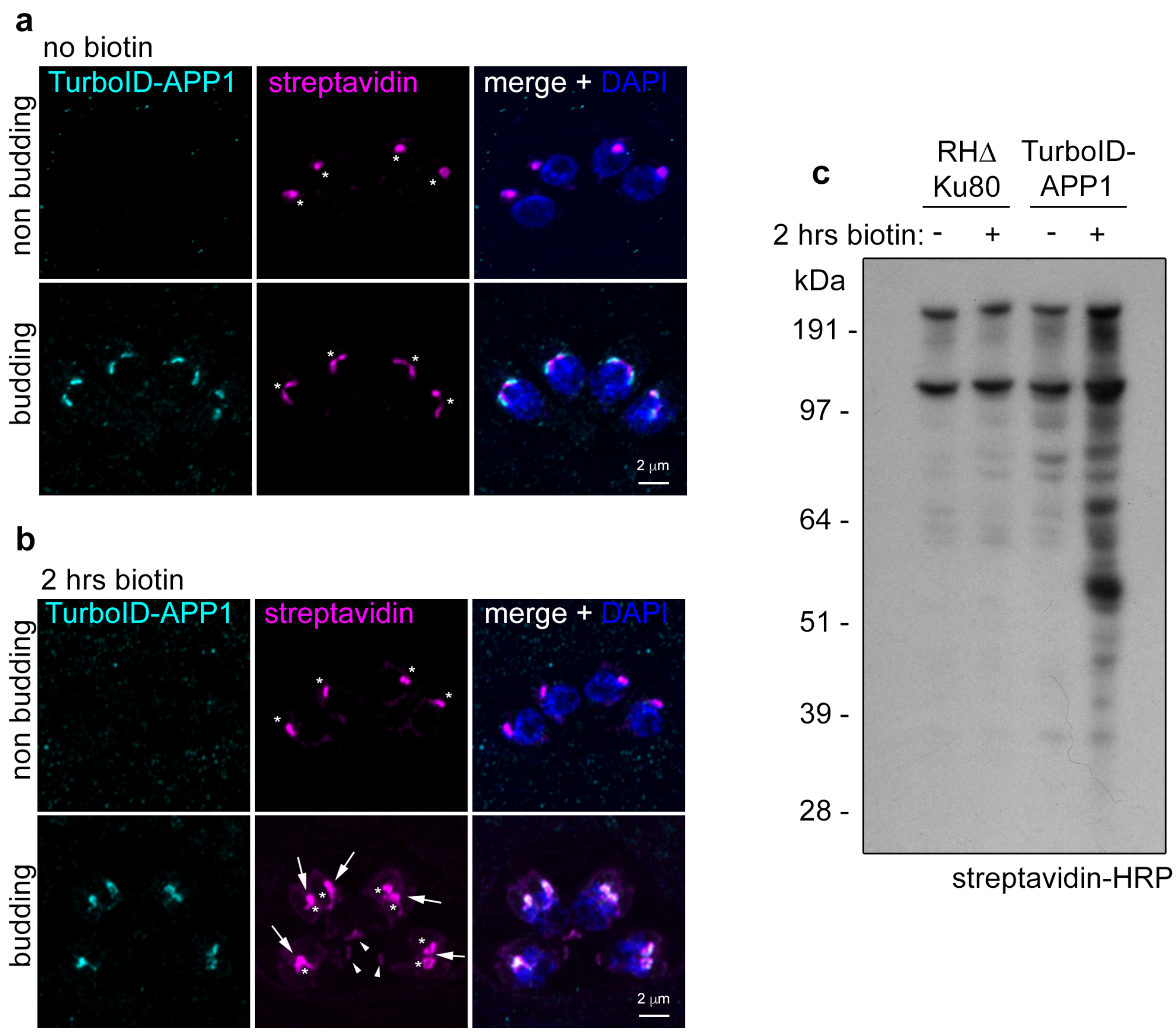


**Supplementary Figure 5: Verification of the TurboID-APP1 parasites.**
a: TurboID-APP1 parasites grown over night in absence of additional biotin. TurboID-APP1, visualized via Ty antiserum (cyan), is only expressed in dividing parasites. Streptavidin-A594 is used to visualize biotinylated proteins, which are always present in the apicoplast (asterisks). b: TurboID-APP1 parasites grown over night and treated with additional biotin (500 µM) for 2 hrs before fixation. Streptavidin-A594 visualizes biotinylated proteins. APP1-dependent biotinylation is detected close to the apicoplast and daughter IMC (arrows) and in the basal complex of the mother (arrowheads). Asterisks: apicoplast. DAPI stains DNA. c: Western blot analysis of RHΔKu80 and TurboID-APP1 parasite lysates. Parasites were treated with or without 500 µM biotin for 2 hrs before harvest. Biotinylated proteins were detected using streptavidin coupled to HRP. kDa: molecular size in kilo Daltons. The western blot shown represents a single experiment.

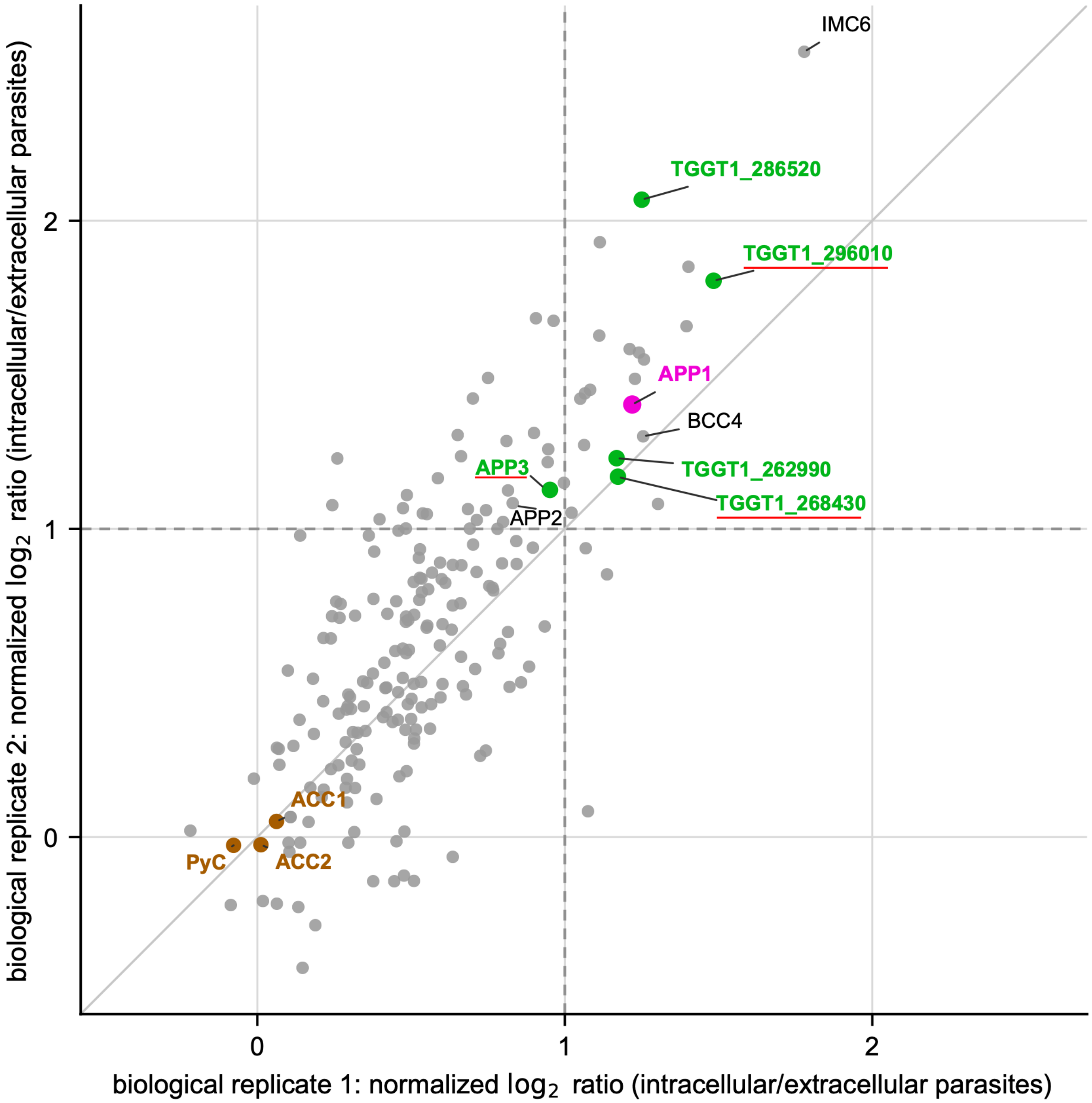


**Supplementary Figure 6. Correlation between two APP1 TurboID biological replicates.** Protein abundance ratios between intracellular and extracellular samples were normalized and $\log_2$ transformed. X-axis depicts proteins identified in APP1 TurboID replicate 1 and y-axis shows proteins identified in APP1 TurboID replicate 2. Dashed lines indicate 2-fold ($\log_2$=1) increase in each replicate; the solid line indicates perfect correlation. Correlation between replicates was assessed using Spearman's rank correlation coefficient based on $\log_2$-transformed normalized abundance ratios ($\rho$ = 0.73; n=204 proteins). Brown lined factors are endogenously biotinylated proteins. Green lined factors are hypothetical and unstudied proteins (red underscored factors were selected for experimental validation). Note that APP2 (TGGT1_313360) was discovered and characterized in the co-submitted study by Mead et al. Source data are provided in the source data file.

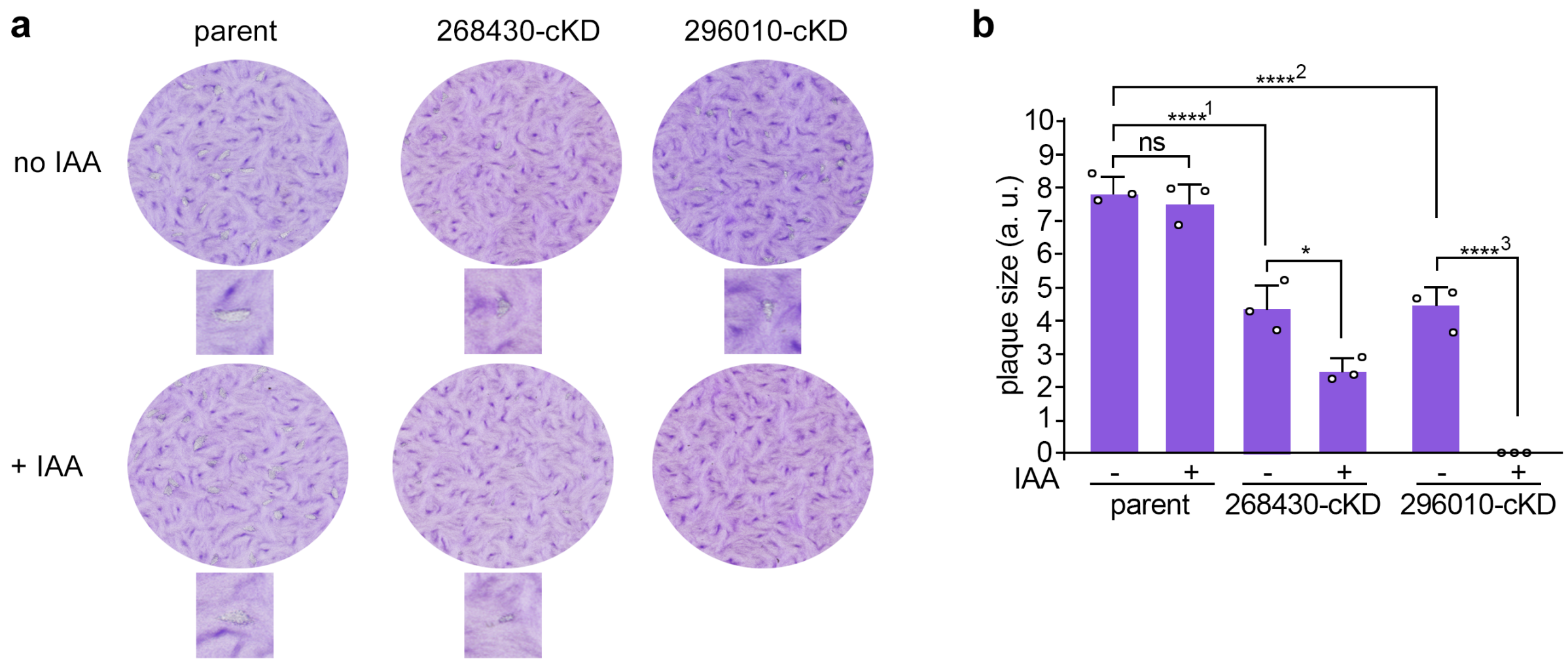


**Supplementary Figure 7. Plaque assays of APP1 BioID candidates.** The ORF of TGGT1_268430 and TGGT1_296010 was tagged at the 5'-end with 5xV5-mAID tag **(**Supplementary Fig 1f&g). a: Representative images of seven-day plaque assays indicating severely reduced parasite fitness for 268430-cKD and 296010-cKD even in absence of IAA. Upon growth in presence of IAA, TGGT1_268430 forms even smaller plaques, while no plaques were observed for TGGT1_296010. Insets show 2.5x zoom in on a single plaque. The parent is Tir1ΔKu80-ALFA-APP1. b: Quantification of plaque size. 50 plaques for each of the n=3 biological replicates were measured. Data represent mean values + std. One-way ANOVA with Tukey's HSD was used to calculate significance. ns= not significant; *: $p$= 0.0167; ****[1]: $p$= $6.34 \times 10^{-5}$, ****[2]: $p$= $7.21 \times 10^{-5}$, ****[3]: $p$= $1.65 \times 10^{-6}$.

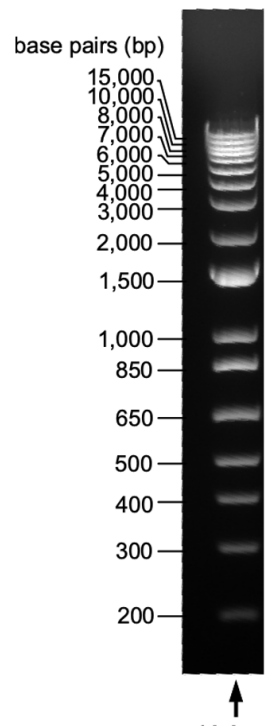


1kb+ ladder

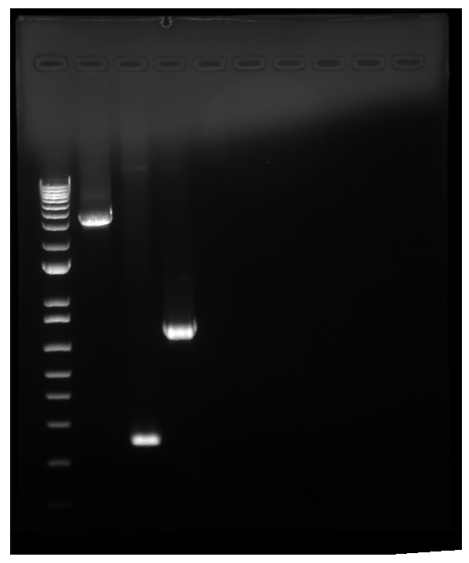

Supp Fig 1a

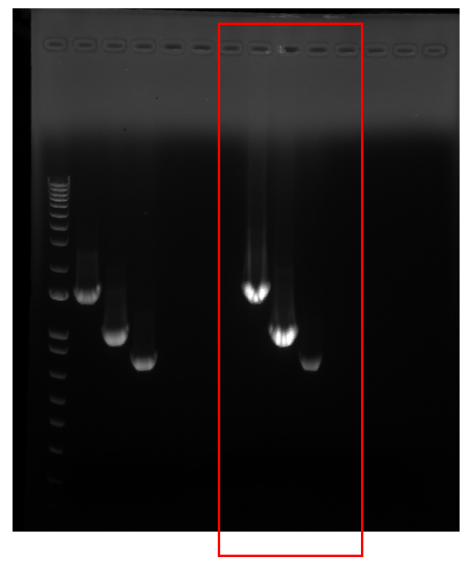

Supp Fig 1b

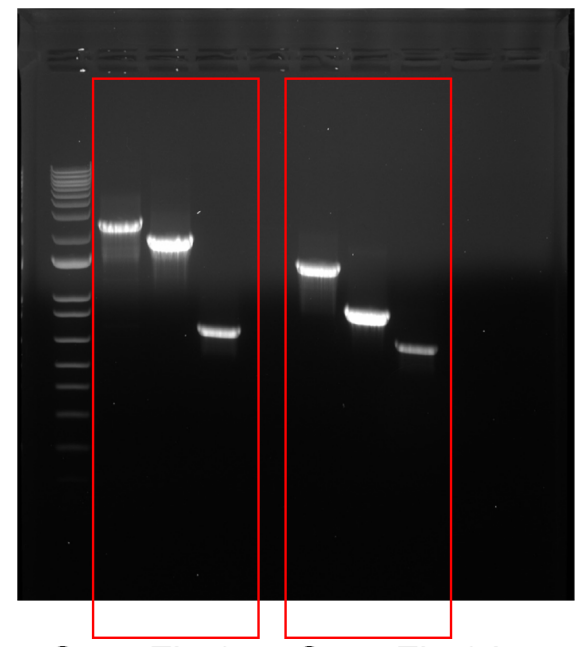

Supp Fig 1c Supp Fig 1d

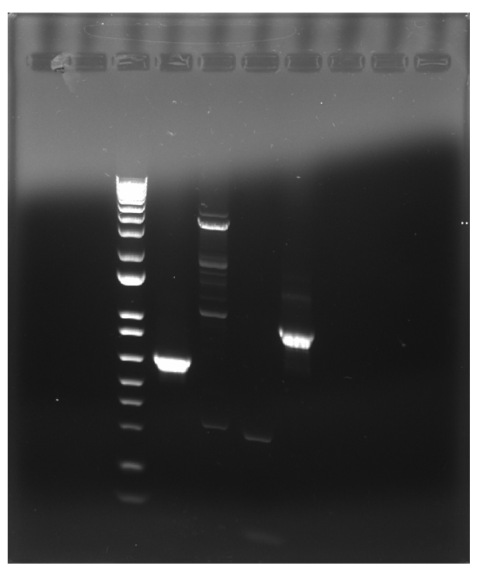

Supp Fig 1e

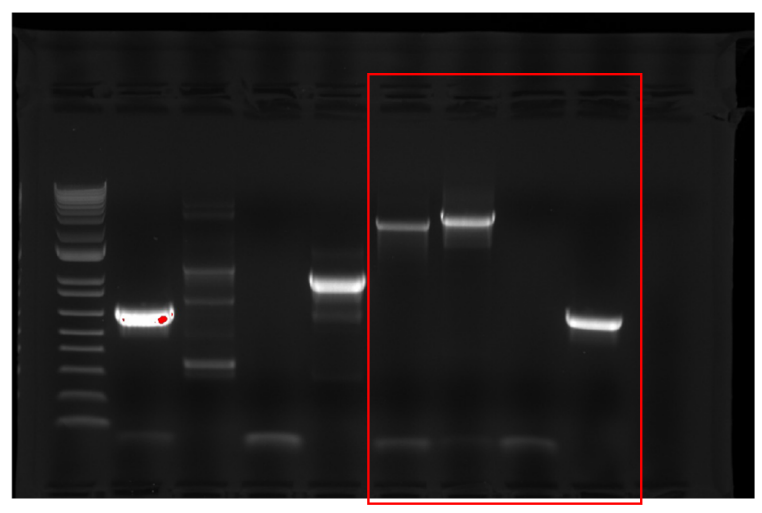

Supp Fig 1f

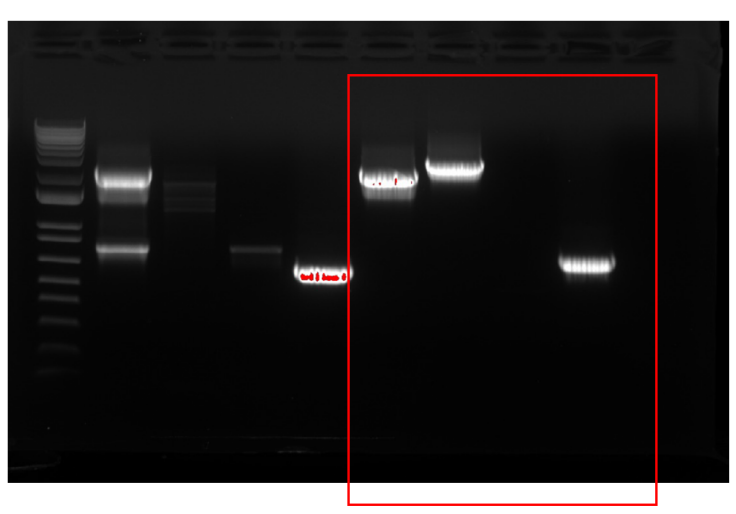

Supp Fig 1g

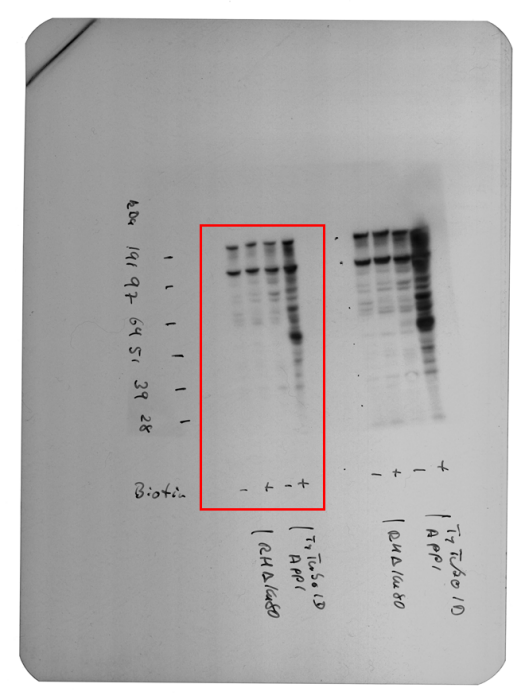

Supp Fig 5c

**Uncropped images of diagnostic PCRs and APP1 TurboID western blot. The** DNA 1 kb+ ladder has the same banding pattern across all agarose gels in **Supplementary Figure 1**.

## Supplementary Table 1: Oligonucleotides used in this study.

| Oligo name | Purpose | Sequence (5'–>3') |
|---|---|---|
| **BCC4_pU6_1_s** | to create 5' end targeting single guide RNA. Has flank to clone into pU6 universal. sgRNA sequence: GGAATGCTGAACGAGCGCTC | AAGTT GGAATGCTGAACGAGCGCTC G |
| **BCC4_pU6_2_as** | 5' end targeting single guide RNA. Has flank to clone into pU6 universal. | AAAAC GAGCGCTCGTTCAGCATTCC A |
| **BCC4_sense** | has 35 pb flank for 5'end integration and binds HXGPRT ORF | TGTTTTCTGCCTCCG CGTTAACTTCTTTTTCTGGAAAAATG GCGTCCAAACCCATTGAA |
| **BCC4_as** | has flank for 5' end integration and binds in the end of TurboID sequence | CAGCGCTGTCGAGCGCAGCGCCCG ATCCACTTCGTTCGTTCAG ctgcagCTTTTCGGCAGACC |
| **APP1_pU6** | to create 5' end targeting single guide RNA. Has flank to clone into pU6 universal. sgRNA sequence: CTCTTCCACAAAAGGTACAA | TCTGGTAAATGGGGATGTCAAGTTgC TCTTCCACAAAAGGTACAAGTTTTAG AGCTAGAAATAGCAAGTT |
| **APP1_s** | to endogenously tag APP1 at 5' end with HX_Ty-TurboID, binds at beginning of HXGPRT ORF | TACCCAGTTATCACTTTTCTCACTCTT CCACAAAA aaaATGGCGTCCAAACCCATTGAA |
| **APP1_s** | for promoter replacement, binds in the beginning of 5'dhfr and ~ 500bp upstream of APP1 ATG | GACAGCCTCTTCGCTTATGGATATGT GTAATAAATCACGAAACCTTGCATTC AAACC |
| **APP1_as** | for promoter replacement, binds in the end of Ty and close behind the APP1 ATG | CGCGAATTCCCTACAAACTCCTCCG GAACGTAATC GTCAAGTGGATCTTGGTTAGTATG |
| **APP1_s** | to test for 5' end integration of DHFR-tet7sag4 in the APP1 locus | TCTTCTTGTGCGTCTCGAGCG |
| **APP1_as** | to test for 5' end integration of DHFR-tet7sag4 in the APP1 locus | CTCTTCTTCACTGCCGCTTGC |
| **APP1_as** | has 35 bp flank for 5' end integration and binds in the end of ALFA sequence | CGCGAATTCCCTACAAACTCCTCCG GAACGTAATCCGGCTCCGTGAGGCG TCGGC |
| **APP1_as** | has 35 bp flank for 5' end integration and binds in the end of TurboID sequence | CGCGAATTCCCTACAAACTCCTCCG GAACGTAATC ctgcagCTTTTCGGCAGACCgc |
| **ATG8pU6-1_as** | to create 5' end targeting single guide RNA. Has flank to clone into pU6 universal. sgRNA sequence: CGATGGCATGTTTTTCAAGA | TCTTGAAAAACATGCCATCGcAACTT GACATCCCCATTTACCAGA |
| **ATG8pU6-2_s** | to create 5' end targeting single guide RNA. Has flank to clone into pU6 universal. | CGATGGCATGTTTTTCAAGAGTTTTA GAGCTAGAAATAGCAAGTT |
| **ATG8-DHFR-s** | has 35 bp flank for 5' end integration and binds in the beginning of DHFR ORF | AAAACCCACCAAACCAGATATTTCGC CGTCTTGAAAaaaATGCAGAAACCGG TGTGTCTG |
| **ATG8-mAID-as** | has 35 bp flank 5' end integration and binds in the linker sequence after the mAID sequence | AAGCTCACCGAAGGACACTTCGTCG CGAATCGATGGcagctgggtcgagcccg |
| **PIK4II (TGGT1_296010) pU6_1_as** | to create 5'end targeting single guide RNA. Has flank to clone into pU6 universal. sgRNA sequence: TGCCATCGTGGGTCCTCCCG | CGGGAGGACCCACGATGGCA c AACTTGACATCCCCATTTACCAGA |
| **PIK4II (TGGT1_296010) pU6_2_s** | to create 5' end targeting single guide RNA. Has flank to clone into pU6 universal. | gTGCCATCGTGGGTCCTCCCG GTTTTAGAGCTAGAAATAGCAAGTT |

| | | |
|---|---|---|
| **PIK4II (TGGT1_296010) s** | has 35bp flank for 5' integration and binds at the Start of DHFR ORF | CCACCAAATTGGCTTCGGGCGCCGC GGGAGGACCCAAAATGCAGAAACCG GTGTGTCTG |
| **PIK4II (TGGT1_296010) as** | has 35bp flank for 5' integration and binds linker sequence after mAID sequence | ACACGCTCTATTGGTGTGGGCGAGG ACAGCGATGC cagctgggtcgagcccgagcc |
| **PIK4II (TGGT1_296010) s** | to test for 5'end replacement | TTCCGTCAAAGGCGTCCCG |
| **PIK4II (TGGT1_296010) as** | to test for 5'end replacement | TTTGCCACCGTCGGGTCTCG |
| **TGGT1_268430_pU6_1_as** | to create 5'end targeting single guide RNA guide. Has flank to clone into pU6 universal. sgRNA sequence: CCTCGATACTTTTCGATGG | aac CCATCGAAAAGATATCGAGG c AACTTGACATCCCCATTTACCAGA |
| **TGGT1_268430_pU6_2_s** | to create 5'end targeting single guide RNA guide. Has flank to clone into pU6 universal. | g CCTCGATATCTTTTCGATGG GTTTTAGAGCTAGAAATAGCAAGTT |
| **TGGT1_268430_s** | has 35bp flank for 5' integration and binds at the Start of DHFR ORF | ACTGTAAACTATCACGTGCGTTCCTC GATATCTTT AAAATGCAGAAACCGGTGTGTCTG |
| **TGGT1_268430_as** | has 35bp flank for 5' integration and binds linker sequence after mAID sequence | TGGGGGGCTCCTTGGACATAATCAT TGACTCCCGCcagctgggtcgagcccgagcc |
| **TGGT1_268430_s** | to test for 5'end replacement | TTCGTAACAGCTGGCTGAAGCG |
| **TGGT1_268430_as** | to test for 5'end replacement | AAGGACGCTTCTTCGGTAGACTCG |
| **APP3 (TGGT1_221280)_pU6_1_as** | to create 3'end targeting single guide RNA guide. Has flank to clone into pU6 universal. sgRNA sequence: TTCTTCAGACCCTGTTAACA | TGTTAACAGGGTCTGAAGAA c AACTTGACATCCCCATTTACCAGA |
| **APP3 (TGGT1_221280)_pU6_2_s** | to create 3'end targeting single guide RNA guide. Has flank to clone into pU6 universal. | g TTCTTCAGACCCTGTTAACA GTTTTAGAGCTAGAAATAGCAAGTT |
| **APP3 (TGGT1_221280)** | has 35bp flank for 3' integration and binds at the linker in front of the mAID sequence | CTGCTCGAGTTCCAAAGCCC TTa TTg ACg GGG TCT GAA GAA GGCGGAGGTGGCTCGgaga |
| **APP3 (TGGT1_221280)** | has 35bp flank for 3' integration and binds at the 3'region of the DHFR cassette | GACGGCTTTCTCTTTCCTCTCGCTGT CTTAAAGAA CATCCTGCAAGTGCATAGAAGG |
| **APP3 (TGGT1_221280)** | to test for 3'end replacement | GCAGCAGAAAGCTCCGACCG |
| **APP3 (TGGT1_221280)** | to test for 3' end replacement | TCGCTTTTGAGGCAGCAGTCC |
| **AID_as** | to verify 3''end replacement with mAID | AGACCATCACGTTCTTGCGG |
| **DHFR_s** | to verify 5' end replacement with DHFR ORF | TCGTGCATCATGTATCAGCGG |
| **tet7sag4_s** | to verify promoter replacement with tet7sag4 | AGCATACTGCAACTGCTTTCG |
| **HXGPRT_s** | to verify 5' replacement with HXGPRT ORF | GGGAACAAAAGCTGGGTACCAAAAT GGCGTCCAAACCCATTGAA |
| **actin_s** | for qPCR, to quantify in nuclear genome | GGGACGACATGGAGAAAATC |
| **actin_as** | for qPCR, to quantify nuclear genome | AGAAAGAACGGCCTGGATAG |
| **TogoCr29_s** | for qPCR, to quantify apicoplast genome | GCCCGTACTAAAACTGACACAAG |
| **TogoCr29_as** | for qPCR, to quantify apicoplast genome | AGGCATCCTTTATCCCGAAG |

**Supplementary Table 2: Antisera used in this study.**

**Primary antisera**

| Name | Species | Source and validation reference | Dilution |
|---|---|---|---|
| **ALFA** | rabbit | Synaptic Systems (N1583) [1] | PS-ExM: 1:1000; pan-ExM: 1:400 |
| **ALFA** | guinea pig | Synaptic Systems (N1584) [1] | PS-ExM: 1:1000; pan-ExM: 1:400 |
| **CrCentrin (Cen20H5) (centrosome)** | mouse | Millipore-Sigma (ZMS1054) [2, 3] | IFA/PS-ExM: 1:500 |
| **$F_1$B ATPase (5F4) (mitochondrion)** | mouse | Peter Bradley [4, 5] | IFA: 1:500 |
| **IMC3(aa1-120)** | rabbit | In house [6] | IFA/PS-ExM: 1:1000 |
| **SRTL (Golgi)** | rat | Stanislas Tomavo [7] | IFA: 1:300 |
| **α-tubulin (12G10)** | mouse | Developmental Studies Hybridoma Bank [1, 8, 9] | pan-ExM: 1:250 |
| **Ty-1 (BB2)** | mouse | Sebastian Lourido [10, 11] | IFA: 1:500 |
| **V5 (SV5-P-K)** | rabbit | Abcam (#206566) [12] | PS-ExM: 1:500; pan-ExM: 1:300 |
| **V5 (SV5-PK1)** | mouse | Bio-Rad (#MCA1360GA) [12, 13] | PS-ExM: 1:500; pan-ExM: 1:300 |
| **MORN1 (UGA54)** | rabbit | Boris Striepen [14] | pan-ExM: 1:300 |

**Secondary antisera**

| Name | Species | Source |
|---|---|---|
| **Goat anti-mouse Alexa488/Alexa594** | goat | Invitrogen (#A-11001, Alexa488; #A-11005, Alexa594) |
| **Goat anti-guinea pigALEXA488** | goat | Invitrogen (#A-11073) |
| **Goat anti-rabbit Alexa488/Alexa594/Alexa647** | goat | Invitrogen(#A11008, Alexa488; #A-11012, Alexa594; #A-21245, Alexa647) |
| **Goat anti-mouse DyLight488** | goat | ThermoFisher (#35503) |
| **Goat anti-rabbit DyLight594** | goat | ThermoFisher (#35561) |
| **Goat anti-ratAlexa488** | goat | Invitrogen (#A-11006) |

**Dilutions for all secondary antisera: IFA/PS-ExM: 1:500; pan-ExM: 1:200**

**Protein stain**

| Name | Species | Source | Dilutions |
|---|---|---|---|
| **streptavidin-Alexa488/Alexa594** | - | ThermoFisher(#S11223, Alexa488; #S32356, Alexa594) | IFA/PS-ExM/pan-ExM: 1:1000 |
| **NHS-esterATTO488** | - | SigmaAlderich(#41698) | pan-ExM: 20 μg/ml |
| **Streptavidin-HRP** | - | Invitrogen (#911) | western blot: 1:10000 |

**Supplementary References:**


1. Engelberg, K., Bauwens, C., Ferguson, D. J. P. & Gubbels, M. J. Co-dependent formation of the Toxoplasma gondii subpellicular microtubules and inner membrane skeleton. *MBio* **16**, e0138925 (2025).
2. Tran, J. Q.*, et al.* RNG1 is a late marker of the apical polar ring in Toxoplasma gondii. *Cytoskeleton (Hoboken)* **67**, 586-598 (2010).
3. Wall, R. J.*, et al.* SAS6-like protein in Plasmodium indicates that conoid-associated apical complex proteins persist in invasive stages within the mosquito vector. *Sci Rep* **6**, 28604 (2016).
4. Jacobs, K., Charvat, R. & Arrizabalaga, G. Identification of Fis1 Interactors in Toxoplasma gondii Reveals a Novel Protein Required for Peripheral Distribution of the Mitochondrion. *MBio* **11**, e02732-02719 (2020).
5. Pasquarelli, R. R.*, et al.* Characterization and functional analysis of Toxoplasma Golgi-associated proteins identified by proximity labeling. *MBio* **15**, e0238024 (2024).
6. Engelberg, K.*, et al.* The apical annuli of Toxoplasma gondii are composed of coiled-coil and signalling proteins embedded in the inner membrane complex sutures. *Cell Microbiol* **22**, e13112 (2020).
7. Sloves, P. J.*, et al.* Toxoplasma sortilin-like receptor regulates protein transport and is essential for apical secretory organelle biogenesis and host infection. *Cell Host Microbe* **11**, 515-527 (2012).
8. Jerka-Dziadosz, M.*, et al.* Cellular polarity in ciliates: persistence of global polarity in a disorganized mutant of Tetrahymena thermophila that disrupts cytoskeletal organization. *Dev Biol* **169**, 644-661 (1995).
9. Gubbels, M. J.*, et al.* Toxoplasma gondii's Basal Complex: The Other Apicomplexan Business End Is Multifunctional. *Front Cell Infect Microbiol* **12**, 882166 (2022).
10. Sidik, S. M.*, et al.* A Genome-wide CRISPR Screen in Toxoplasma Identifies Essential Apicomplexan Genes. *Cell* **166**, 1423-1435 e1412 (2016).
11. Bastin, P., Bagherzadeh, Z., Matthews, K. R. & Gull, K. A novel epitope tag system to study protein targeting and organelle biogenesis in Trypanosoma brucei. *Mol Biochem Parasitol* **77**, 235-239 (1996).
12. Engelberg, K., Bechtel, T., Michaud, C., Weerapana, E. & Gubbels, M. J. Proteomic characterization of the Toxoplasma gondii cytokinesis machinery portrays an expanded hierarchy of its assembly and function. *Nat Commun* **13**, 4644 (2022).
13. Rudlaff, R. M., Kraemer, S., Streva, V. A. & Dvorin, J. D. An essential contractile ring protein controls cell division in Plasmodium falciparum. *Nat Commun* **10**, 2181 (2019).
14. Gubbels, M. J., Vaishnava, S., Boot, N., Dubremetz, J. F. & Striepen, B. A MORN-repeat protein is a dynamic component of the Toxoplasma gondii cell division apparatus. *J Cell Sci* **119**, 2236-2245 (2006).
