# Supplementary material for: A dedicated partitioning machinery ensures faithful inheritance of the apicoplast in Toxoplasma gondii
Source: Nat Commun. 2026 Oct 5;17:10368. doi: 10.1038/s41467-026-78103-w (PMC13639030; doi:10.1038/s41467-026-78103-w)
Supplement: Supplementary file 2 [file 41467_2026_78103_MOESM7_ESM.pdf]

nature portfolio

Corresponding author(s): Klemens Engelberg

Last updated by author(s): Aug 28, 2026

Reporting Summary

Nature Portfolio wishes to improve the reproducibility of the work that we publish. This form provides structure for consistency and transparency in reporting. For further information on Nature Portfolio policies, see our Editorial Policies and the Editorial Policy Checklist.

Statistics

For all statistical analyses, confirm that the following items are present in the figure legend, table legend, main text, or Methods section.

n/a	Confirmed	
☐	☒	The exact sample size (*n*) for each experimental group/condition, given as a discrete number and unit of measurement
☐	☒	A statement on whether measurements were taken from distinct samples or whether the same sample was measured repeatedly
☐	☒	The statistical test(s) used AND whether they are one- or two-sided<br>*Only common tests should be described solely by name; describe more complex techniques in the Methods section.*
☒	☐	A description of all covariates tested
☐	☒	A description of any assumptions or corrections, such as tests of normality and adjustment for multiple comparisons
☐	☒	A full description of the statistical parameters including central tendency (e.g. means) or other basic estimates (e.g. regression coefficient) AND variation (e.g. standard deviation) or associated estimates of uncertainty (e.g. confidence intervals)
☐	☒	For null hypothesis testing, the test statistic (e.g. *F*, *t*, *r*) with confidence intervals, effect sizes, degrees of freedom and *P* value noted<br>*Give P values as exact values whenever suitable.*
☒	☐	For Bayesian analysis, information on the choice of priors and Markov chain Monte Carlo settings
☒	☐	For hierarchical and complex designs, identification of the appropriate level for tests and full reporting of outcomes
☒	☐	Estimates of effect sizes (e.g. Cohen's *d*, Pearson's *r*), indicating how they were calculated

Our web collection on statistics for biologists contains articles on many of the points above.

Software and code

Policy information about availability of computer code

Data collection	Imaging:<br>Zen 2.3 (black&blue edition)<br>DeltaVision AcquireUltra-1.2.3
Data analysis	Mass spectrometry:<br>RawConverter 1.1.0.20<br>SeQUESTv3.0<br>DTASelectv2.0<br>SAINTexpressv3.6.3<br>Thermo Proteome Dicoverer v2.4<br>Microsoft 365 suite<br>Imaging:<br>Zen 2.3 (blue edition), FIJ/ImageJ2I(version2.16.0/1.54p)<br>Statistics:<br>R statistical software/R studio (version 2026.01.0+392), R package ggplot2 (ggplot2, version 3.3.3(, R package rstatix, (version 0.7.0)

For manuscripts utilizing custom algorithms or software that are central to the research but not yet described in published literature, software must be made available to editors and reviewers. We strongly encourage code deposition in a community repository (e.g. GitHub). See the Nature Portfolio guidelines for submitting code & software for further information.

Data

Policy information about availability of data

All manuscripts must include a data availability statement. This statement should provide the following information, where applicable:

- Accession codes, unique identifiers, or web links for publicly available datasets
- A description of any restrictions on data availability
- For clinical datasets or third party data, please ensure that the statement adheres to our policy

All relevant data are included in the main text of the manuscript and the supplementary material. Raw data points underlying graphs are present in the Source data file accompanying the manuscript.The mass spectrometry proteomics data have been deposited to the ProteomeXchange Consortium via the Pride partner repository with the dataset identifier PXD075456 (https://www.ebi.ac.uk/pride/archive/projects/PXD075456) .

Research involving human participants, their data, or biological material

Policy information about studies with human participants or human data. See also policy information about sex, gender (identity/presentation), and sexual orientation and race, ethnicity and racism.

Reporting on sex and gender	n/a
Reporting on race, ethnicity, or other socially relevant groupings	n/a
Population characteristics	n/a
Recruitment	n/a
Ethics oversight	n/a

Note that full information on the approval of the study protocol must also be provided in the manuscript.

Field-specific reporting

Please select the one below that is the best fit for your research. If you are not sure, read the appropriate sections before making your selection.

☒ Life sciences ☐ Behavioural & social sciences ☐ Ecological, evolutionary & environmental sciences

For a reference copy of the document with all sections, see nature.com/documents/nr-reporting-summary-flat.pdf

Life sciences study design

All studies must disclose on these points even when the disclosure is negative.

Sample size	No sample size calculations were performed. Unless stated otherwise, all experiments were performed with n=3 biological replicates to allow statistical analyses. For quantification of apicoplast partitioning defects, 100 vacuoles per replicate were analyzed. For quantification of Golgi, centrosome, and mitochondrion inheritance defects, 50 vacuoles per replicate were analyzed. Quantifications of centrosome mispositioning (Fig 8 f) are based on 50 vacuoles, harboring dividing parasites, per replicate. Quantification of APP1 mislocalization in PS-ExM (Fig 8e) is based on a total of 90 daughter cells per condition, divided over n=3 biological replicates.
Data exclusions	The authors declare that no data were excluded.
Replication	Experiments were executed as biological replicates, measuring the same clonal line on different days. Unless stated otherwise, three biological replicates were used. BioID experiments were done as two biological replicates, which were split into technical replicates for the MS run. All experiments resulted in comparable results.
Randomization	Randomization was not applied. Knockdown/protein-depletion and non-induced control samples were treated under identical conditions and processed side by side.
Blinding	Except for the experiment shown in Fig 8e, investigators were blinded to the identity of the samples for all other microscopy-based quantifications. Sample identities were revealed only after the analyses were completed. For BioID samples, investigators were not blinded since these experiments reached quantitative endpoints, which are less likely to be influenced by investigator bias.

Behavioural & social sciences study design

All studies must disclose on these points even when the disclosure is negative.

Study description	n/a
Research sample	n/a
Sampling strategy	n/a
Data collection	n/a
Timing	n/a
Data exclusions	n/a
Non-participation	n/a
Randomization	n/a

Ecological, evolutionary & environmental sciences study design

All studies must disclose on these points even when the disclosure is negative.

Study description	n/a
Research sample	n/a
Sampling strategy	n/a
Data collection	n/a
Timing and spatial scale	n/a
Data exclusions	n/a
Reproducibility	n/a
Randomization	n/a
Blinding	n/a

Did the study involve field work? ☐ Yes ☒ No

Field work, collection and transport

Field conditions	n/a
Location	n/a
Access & import/export	n/a
Disturbance	n/a

Reporting for specific materials, systems and methods

We require information from authors about some types of materials, experimental systems and methods used in many studies. Here, indicate whether each material, system or method listed is relevant to your study. If you are not sure if a list item applies to your research, read the appropriate section before selecting a response.

Materials & experimental systems

n/a	Involved in the study
☐	☒ Antibodies
☐	☒ Eukaryotic cell lines
☒	☐ Palaeontology and archaeology
☒	☐ Animals and other organisms
☒	☐ Clinical data
☒	☐ Dual use research of concern
☒	☐ Plants

Methods

n/a	Involved in the study
☒	☐ ChIP-seq
☒	☐ Flow cytometry
☒	☐ MRI-based neuroimaging

Antibodies

Antibodies used

Primary antibodies:
-rabbit anti-ALFA (Synatic Systems, #N1583) and guinea pig anti-ALFA (Synaptic Systems, #N1584). Dilutions: PS-ExM: 1:1000; pan-ExM: 1:400.
-mouse anti-CrCentrin (Cen20H5), Millipore-Sigma, #ZMS1054. Dilutions: IFA&PS-ExM: 1:500.
-mouse anti-F1B ATPase (5F4), kind gift of Peter Bradley. Dilution: IFA: 1:500.
-rabbit anti-IMC3(aa1-120), in house. Dilutions: IFA&PS-ExM: 1:1000.
-rat anti-SRTL, kind gift of Stanislas Tomavo. Dilution: IFA: 1:300.
-mouse anti-alphaTubulin(12G10), Developmental Studies Hybridoma Bank, RRID: AB_1157911. Dilution: pan-ExM: 1:250.
-mouse anti-Ty1 (BB2), kind gift of Sebastian Lourido. Dilution: IFA: I:500.
-rabbit anti-V5 (SV5-P-K), Abcam,#206566. Dilutions: PS-ExM: 1:500; pan-ExM: 1:300.
-mouse anti-V5 (SV5-PK1), Bio-Rad, #MCA1360GA. Dilution: PS-ExM: 1:500; pan-ExM: 1:300.
-rabbit anti-MORN1 (UGA54), kind gift of Boris Striepen. Dilution: pan-ExM: 1:300.

Protein stain:
-sreptavidin-Alexa488: #S11223 and Alexa594: #S322356, ThermoFisher. Dilutions: IFA, PS-ExM and pan-ExM: 1:1000.
-NHS-esterATTO488, SigmaAlderich, #41698. Dilutions: pan-ExM: 20ug/mL.

Secondary Antibodies:
-goat anti-mouse (Alexa488: #A-11001; Alexa594: #A-110054) Invitrogen.
-goat anti-guinea pig Alexa488 (invitrogen, #A-11073).
-goat anti-rabbit (Alexa488: #A11008; Alexa594: #A-11012; Alexa647: #A-21245) Invitrogen.
-goat anti-mouseDylight488, (ThermoFisher, #35503).
-goat anti-rabbitDyLight594, (ThermoFisher, #35561).
-goat anti-ratAlexa488, (Invitrogen, #A-11006)
Dilutions for all secondaries: IFA&PS-ExM: 1:500, pan-ExM: 1:200.

Western blot:
-stretavidin-HRP, (Invitrogen, #S911). Dilution: 1:10000

Validation

The primary antibodies have been validated by the manufacturer and in the following studies, among others:

-rabbit anti-ALFA and guinea pig anti-ALFA : validated for IFA and western blot in human cells, according to manufacturer's website (https://sysy.com/product/N1583#list, https://sysy.com/product/N1584). Previously used in T. gondii: PMID: 40801525
-mouse anti-CrCentrin (Cen20H5): validated for IFA and western blot in human cells, according to manufacturer's website (https://www.sigmaaldrich.com/US/en/product/sigma/zms1054). Previously used in Plasmodium: PMID: 27339728 and in T. gondii: PMID: 20658557
-mouse anti-F1B ATPase (5F4): Previously used in T. gondii: PMID: 32047127 and PMID:39345210
-rabbit anti-IMC3(aa1-120): Previously used in T. gondii: PMID: 31470470
-rat anti-SRTL: Previously used in T. gondii: PMID: 22607804
-mouse anti-alphaTubulin(12G10): reactive against T. gondii, validated for western blot and IFA according to providers website: [https://dshb.biology.uiowa.edu/12G10-anti-alpha-tubulin?quantity=1&product-form=2] PMID: 7781905. Previously used in T. gondii: PMID: 35573773 and PMID: 40801525
-mouse anti-Ty1 (BB2):PMID: 8813669 and previously used in T. gondii: PMID: 27594426
-rabbit anti-V5 (SV5-P-K) and mouse anti-V5 (SV5-PK1): Previously used in Plasmodium: PMID: 31097714 and in T. gondii: PMID: 35941170
-rabbit anti-MORN1 (UGA54): Previously used in T. gondii: PMID: 16684814

Eukaryotic cell lines

Policy information about cell lines and Sex and Gender in Research

Cell line source(s)

human foreskin fibroblasts (HFF:ATCC #CRL-1634, BJ1-hTERT: RRID:CVCL_6573)
T. gondii RHΔKu80: PMID: 19218426
T. gondii TATiΔKu80ΔHXGPRT: kind gift of Lilach Sheiner and based on TATiΔKu80 parasites. PMID: 22144892
T. gondii TirlΔKu80: PMID: 28465425

Authentication

T. gondii cell lines were authenticated for their particular biological function (e.g. high rate of homologous recombination in

Authentication	RHΔKu80 and functionality of Tir1 or Tet-transactivator in Tir1ΔKu80/TATiΔKu80ΔHXGPRT parasites). HFF cells were authenticated at he source and are periodically inspected by microscopy (observation of morphology).
Mycoplasma contamination	HFF/BJ1-hTERT and T. gondii parental lines are periodically tested for Mycoplasma contaminations, in which they tested negative. Established transgenic parasite lines have not been tested.
Commonly misidentified lines (See ICLAC register)	none

Palaeontology and Archaeology

Specimen provenance	n/a
Specimen deposition	n/a
Dating methods	n/a

☐ Tick this box to confirm that the raw and calibrated dates are available in the paper or in Supplementary Information.

Ethics oversight	n/a

Note that full information on the approval of the study protocol must also be provided in the manuscript.

Animals and other research organisms

Policy information about studies involving animals; ARRIVE guidelines recommended for reporting animal research, and Sex and Gender in Research

Laboratory animals	n/a
Wild animals	n/a
Reporting on sex	n/a
Field-collected samples	n/a
Ethics oversight	n/a

Note that full information on the approval of the study protocol must also be provided in the manuscript.

Clinical data

Policy information about clinical studies
All manuscripts should comply with the ICMJE guidelines for publication of clinical research and a completed CONSORT checklist must be included with all submissions.

Clinical trial registration	n/a
Study protocol	n/a
Data collection	n/a
Outcomes	n/a

Dual use research of concern

Policy information about dual use research of concern

Hazards

Could the accidental, deliberate or reckless misuse of agents or technologies generated in the work, or the application of information presented in the manuscript, pose a threat to:

No	Yes	
☒	☐	Public health
☒	☐	National security
☒	☐	Crops and/or livestock
☒	☐	Ecosystems
☒	☐	Any other significant area

Experiments of concern

Does the work involve any of these experiments of concern:

No	Yes	
☒	☐	Demonstrate how to render a vaccine ineffective
☒	☐	Confer resistance to therapeutically useful antibiotics or antiviral agents
☒	☐	Enhance the virulence of a pathogen or render a nonpathogen virulent
☒	☐	Increase transmissibility of a pathogen
☒	☐	Alter the host range of a pathogen
☒	☐	Enable evasion of diagnostic/detection modalities
☒	☐	Enable the weaponization of a biological agent or toxin
☒	☐	Any other potentially harmful combination of experiments and agents

Plants

Seed stocks	n/a
Novel plant genotypes	n/a
Authentication	n/a

ChIP-seq

Data deposition

☐ Confirm that both raw and final processed data have been deposited in a public database such as GEO.

☐ Confirm that you have deposited or provided access to graph files (e.g. BED files) for the called peaks.

Data access links<br>*May remain private before publication.*	n/a
Files in database submission	n/a
Genome browser session<br>(e.g. UCSC)	n/a

Methodology

Replicates	n/a
Sequencing depth	n/a
Antibodies	n/a
Peak calling parameters	n/a
Data quality	n/a
Software	n/a

Flow Cytometry

Plots

Confirm that:

- [ ] The axis labels state the marker and fluorochrome used (e.g. CD4-FITC).
- [ ] The axis scales are clearly visible. Include numbers along axes only for bottom left plot of group (a 'group' is an analysis of identical markers).
- [ ] All plots are contour plots with outliers or pseudocolor plots.
- [ ] A numerical value for number of cells or percentage (with statistics) is provided.

Methodology

Sample preparation	n/a
Instrument	n/a
Software	n/a
Cell population abundance	n/a
Gating strategy	n/a

- [ ] Tick this box to confirm that a figure exemplifying the gating strategy is provided in the Supplementary Information.

Magnetic resonance imaging

Experimental design

Design type	n/a
Design specifications	n/a
Behavioral performance measures	n/a

Acquisition

Imaging type(s)	n/a
Field strength	n/a
Sequence & imaging parameters	n/a
Area of acquisition	n/a

Diffusion MRI ☐ Used ☐ Not used

Preprocessing

Preprocessing software	n/a
Normalization	n/a
Normalization template	n/a
Noise and artifact removal	n/a
Volume censoring	n/a

Statistical modeling & inference

Model type and settings	n/a
Effect(s) tested	n/a

Specify type of analysis: ☐ Whole brain ☐ ROI-based ☐ Both

Statistic type for inference
(See Eklund et al. 2016)

n/a

Correction

n/a

Models & analysis

n/a	Involved in the study
☐	☐ Functional and/or effective connectivity
☐	☐ Graph analysis
☐	☐ Multivariate modeling or predictive analysis

Functional and/or effective connectivity

n/a

Graph analysis

n/a

Multivariate modeling and predictive analysis

n/a
