# Supplementary material for: A dedicated partitioning machinery ensures faithful inheritance of the apicoplast in Toxoplasma gondii
Source: Nat Commun. 2026 Oct 5;17:10368. doi: 10.1038/s41467-026-78103-w (PMC13639030; doi:10.1038/s41467-026-78103-w)
Supplement: Supplementary file 3 [file 41467_2026_78103_MOESM8_ESM.pdf]

nature portfolio

Peer Review File

A dedicated partitioning machinery ensures faithful inheritance of the apicoplast in *Toxoplasma gondii*

Corresponding Author: Dr Klemens Engelberg

This file contains all reviewer reports in order by version, followed by all author rebuttals in order by version.

Version 0:

Reviewer comments:

Reviewer #1

(Remarks to the Author)
In “A dedicated partitioning machinery ensures faithful inheritance of the apicoplast in T. gondii,” Eichlin and colleagues identify two proteins, APP1 And APP3 involved in apicoplast partitioning. Using expansion microscopy, the authors observe that APP1 anchors the apicoplast to the daughter cell scaffold during cell division. Then they identify APP3, a potential interacting partner of APP1 via a proximity biotinylation approach. Interestigly, APP3 is required to position APP1 on the basal complex indicating that both proteins interact and that the interaction is essential for apicoplast partitioning.
Overall, this paper brings novel insights regarding the proteins mediating apicoplast partitioning in T. gondii. Importantly, the authors establish APP1 and APP3 as critical cytoskeleton-anchored facilitators of apicoplast inheritance. The manuscript was very well written and scientifically sound. Expansion microscopy is ideally suited for observing apicoplast partitioning. Importantly, the study maintains a high degree of rigor, with robust sample sizes across a standard level of biological replicates.
Perhaps the biggest concern about the results of this manuscript is that there is no biochemical evidence that APP1 and APP3 physically interact with each other. Similarly, they mention that ATG8 could be directly interacting with APP1. While this is only a hypothesis, validating a direct APP1/APP3 interaction will provide mechanistic insight to the manuscript, significantly strengthening the paper. Both APP1 and APP3 are extremely large, and an immunoprecipitation might not be possible without considerable troubleshooting. Therefore the language in the paper should be modified as the only evidence of a potential interaction stems from microscopy observations. Other than that, the authors should provide data (IFA and a WB, as supplementary figures) to demonstrate that their APP1-TurboID strain is functional.

Minor concerns:
- It will be good to highlight the tissue cyst forming and non-tissue cyst forming coccidians in the figure 1d.
- Thereis a lack of negative controls for the IFAs and the expansion microscopy. Is there are reason as to why that was not included?
- Please divide the supplementary figure using letters (A, B, C...) otherwise it is hard to follow.
- Why was the 5'end chosen of APP1 chosen? Did you also try the 3 end?
- It is unclear when does the IMC3 recruitment happen. I think this should be mentioned in the figure and also in the figure 2b schematic.
- The authors should choose a different title for figure 3 (“APP1 depletion disrupts apicoplast partitioning”) as the authors don’t examine apicoplast partitioning in this figure.
- Figure 4b does not state how many vacuoles were counted. Then, in figure 4c, the authors measure apicoplast loss with qPCR. The control strain used is the parental strain (TATiΔHXGPRT), but shouldn’t the authors use the conditional knockdown strain minus ATc as a control? If not, can the authors explain why? In addition, why did the authors express their qPCR data as fold as % of vacuoles rather than fold expression?
- Is there an explanation to why arbitrary units were chosen to quantify the plaque assays? Just a brief explanation will suffice.
- There are no arrows in figures 4d, 5b and 5c even though they are indicated in the main manuscript.
- The title for Figure 7 reads, “APP3 is critical for proliferation and localizes to the outer core of the centrosome prior to APP” – the authors should change APP to APP1. In addition, there is a typo for Figure 7f – APP3 is listed twice in the fluorescent channel legend.

Reviewer #2

(Remarks to the Author)
I co-reviewed this manuscript with one of the reviewers who provided the listed reports. This is part of the Nature Communications initiative to facilitate training in peer review and to provide appropriate recognition for Early Career Researchers who co-review manuscripts.

Reviewer #3

(Remarks to the Author)
I co-reviewed this manuscript with one of the reviewers who provided the listed reports. This is part of the Nature Communications initiative to facilitate training in peer review and to provide appropriate recognition for Early Career Researchers who co-review manuscripts.

Reviewer #4

(Remarks to the Author)
This is manuscript describes a very comprehensive characterization of 2 proteins critical for the division of the Toxoplasma apicoplast. The data presented is significant and substantially expands our understanding of the cell biology of this important parasite. There are only a few minor issues that can be addressed before publication.
1. There is a lack of clarity as to the knockdown system used for APP3 depletion. For figure 7 they use the TATi system to test propagation in tissue culture. For figure 8, they switch to the AID system, but do not explain why or show that the AID system recapitulates the plaque phenotype seen in figure 7. More concerning is that in figure 8, some panels (A, F, B and D) use the mAID system other panels use the TATi system (+/- ATc). This needs to be clarified. Ideally, only one knockdown system per figure is presented. Also, for the mAID system, the authors should perform a time course in the presence of IAA to confirm that the protein levels are being depleted and when.
2. Figure 7F: APP3 is labeled twice.
3. Figure 8: Authors should show the staining for both ALPHA-APP1 and APP3-mAID-5xV5 to confirm dual tagging.
4. The summaries for each of the result sessions is a great feature of the paper, but at times it goes beyond summarizing and moves into speculations that are not directly supported by the data in the particular session. It might be better to limit the summaries to a recap of the results and moving the speculation and interpretation to the discussion session.
5. The manuscript by Mead et al does a very good job of referring to this manuscript and discussing similarities and differences. This manuscript should do the same and draw parallels between the two studies.

Reviewer #5

(Remarks to the Author)
I co-reviewed this manuscript with one of the reviewers who provided the listed reports. This is part of the Nature Communications initiative to facilitate training in peer review and to provide appropriate recognition for Early Career Researchers who co-review manuscripts.

Reviewer #6

(Remarks to the Author)
Summary
In this study, Eichlin et al. identify and characterize TGGT1_287970 (APP1) and demonstrate that it functions as a connector between the apicoplast and the daughter cell cytoskeleton during Toxoplasma gondii endodyogeny. Using an elegant combination of genetic perturbations, advanced imaging approaches, and proximity biotinylation, the authors convincingly dissect the mechanistic role of APP1. In addition, they identify a second factor, APP3, and show that APP3 acts upstream to position APP1 at a specific subdomain of the basal complex. Together, these findings define a hierarchical system that links the apicoplast to the cell division machinery. This work therefore represents a conceptual advance, as it identifies a dedicated anchoring machinery underlying faithful apicoplast inheritance.

Own opinion
This is a very strong and well executed study that is important and suitable for publication in Nature Communications. The work addresses a long-standing question in apicomplexan cell biology, providing novel, mechanistic insights by identifying an anchoring apparatus, critical for apicoplast partitioning. A particular strength of the manuscript is the high quality of imaging. The localisation analysis is very beautiful and well executed. Overall the manuscript is very rigurous and my comments are only minor and can be adrdressed relatively easily.

Concerns:
1) Many of the key conclusions rely on representative images rather than quantitative analysis. However, since one central point of the study is the dynamic behaviour of APP1 and APP3, additional quantification would substantially strengthen the manuscript. In particular: The proximity of APP1 to the centrosome and apicoplast should be quantified by distance measurements. Similarly, the asymmetric localization of APP1 to the BC should be quantified.

2) The proposed model relies on dynamic behaviour of APP1 and APP3 during the cell cycle. While the fixed-imaging is of very high quality,the authors could strengthen this aspect by performing live-cell imaging.

3) Line 159: The sentence “we generated a conditional knock-down line by replacing APP1’s promoter with an anhydrous tetracycline (ATc) regulatable TetO7sag4 promoter” could be improved for clarity. I suggest: “We generated a conditional knock-down using the Tet-Transactivation system (TATi).”

4) The discussion appropriately addresses the limited phylogenetic conservation of APP proteins. Expanding this point slightly—particularly in comparison to systems such as PfAnchor in Plasmodium could further strengthen the evolutionary context of the study.

Version 1:

Reviewer comments:

Reviewer #1

(Remarks to the Author)
Thank you for the detailed response. I believe the authors have addressed all the comments from the reviewers, and the manuscript is ready for publication.

Before that, I would like to suggest one minor modification: on figure 5b, the authors should use the same terminology describing endodyogeny as in figures 2 and 4: bud initiation, early budding, mid budding and late budding.

Reviewer #2

(Remarks to the Author)
I co-reviewed this manuscript with one of the reviewers who provided the listed reports. This is part of the Nature Communications initiative to facilitate training in peer review and to provide appropriate recognition for Early Career Researchers who co-review manuscripts.

Reviewer #3

(Remarks to the Author)
I co-reviewed this manuscript with one of the reviewers who provided the listed reports. This is part of the Nature Communications initiative to facilitate training in peer review and to provide appropriate recognition for Early Career Researchers who co-review manuscripts.

Reviewer #4

(Remarks to the Author)
The authors have responded to the reviews appropriately and have strengthened the manuscript in the process.

Reviewer #5

(Remarks to the Author)
I co-reviewed this manuscript with one of the reviewers who provided the listed reports. This is part of the Nature Communications initiative to facilitate training in peer review and to provide appropriate recognition for Early Career Researchers who co-review manuscripts.

Reviewer #6

(Remarks to the Author)
In their revision, the authors provided a detailed response to the reviewers comments and addressed all my concerns. I think the revised manuscript is significantly improved and provides a compelling and rigorous characterization of the machinery governing apicoplast inheritance in Toxoplasma gondii. I have no further comments and recommend the manuscript for publication and congratulate the authors to a very nice study.

Open Access This Peer Review File is licensed under a Creative Commons Attribution 4.0 International License, which permits use, sharing, adaptation, distribution and reproduction in any medium or format, as long as you give appropriate credit to the original author(s) and the source, provide a link to the Creative Commons license, and indicate if changes were made.
In cases where reviewers are anonymous, credit should be given to 'Anonymous Referee' and the source.
The images or other third party material in this Peer Review File are included in the article's Creative Commons license, unless indicated otherwise in a credit line to the material. If material is not included in the article's Creative Commons license and your intended use is not permitted by statutory regulation or exceeds the permitted use, you will need to obtain permission directly from the copyright holder.
To view a copy of this license, visit https://creativecommons.org/licenses/by/4.0/

REVIEWER COMMENTS

Reviewer #1 (Remarks to the Author):

In “A dedicated partitioning machinery ensures faithful inheritance of the apicoplast in T. gondii,” Eichlin and colleagues identify two proteins, APP1 And APP3 involved in apicoplast partitioning. Using expansion microscopy, the authors observe that APP1 anchors the apicoplast to the daughter cell scaffold during cell division. Then they identify APP3, a potential interacting partner of APP1 via a proximity biotinylation approach. Interestigly, APP3 is required to position APP1 on the basal complex indicating that both proteins interact and that the interaction is essential for apicoplast partitioning.

Overall, this paper brings novel insights regarding the proteins mediating apicoplast partitioning in T. gondii. Importantly, the authors establish APP1 and APP3 as critical cytoskeleton-anchored facilitators of apicoplast inheritance. The manuscript was very well written and scientifically sound. Expansion microscopy is ideally suited for observing apicoplast partitioning. Importantly, the study maintains a high degree of rigor, with robust sample sizes across a standard level of biological replicates.

Perhaps the biggest concern about the results of this manuscript is that there is no biochemical evidence that APP1 and APP3 physically interact with each other. Similarly, they mention that ATG8 could be directly interacting with APP1. While this is only a hypothesis, validating a direct APP1/APP3 interaction will provide mechanistic insight to the manuscript, significantly strengthening the paper. Both APP1 and APP3 are extremely large, and an immunoprecipitation might not be possible without considerable troubleshooting. Therefore the language in the paper should be modified as the only evidence of a potential interaction stems from microscopy observations. Other than that, the authors should provide data (IFA and a WB, as supplementary figures) to demonstrate that their APP1-TurboID strain is functional.

We thank the reviewer for the positive assessment of our manuscript. We tried immunoprecipitation of APP1, but as the reviewer points out, the large molecular sizes of APP1 and 3 (molecular mass of 191 and 438 kDa, respectively) interfered with our attempts. We followed the reviewer’s suggestion and adjusted the language throughout the manuscript, leaving a potential direct interaction between APP1, APP3, and ATG8 more ambiguous.

Regarding the TurboID-APP1 strain, we now include the requested verifications of the TurboID-APP1 strain in Supplementary Figure 5.

Minor concerns:

- It will be good to highlight the tissue cyst forming and non-tissue cyst forming coccidians in the figure 1d.

That is a great suggestion, and we have added this labeling accordingly.

- Thereis a lack of negative controls for the IFAs and the expansion microscopy. Is there are reason as to why that was not included?

We provide a negative control for the used V5 and ALFA antisera in pan-ExM in the figure below. Additionally, all antisera used in this study have been extensively used by us and others (see Supplementary Data 2), with no reported discernable cross-reactivity in parental strains.

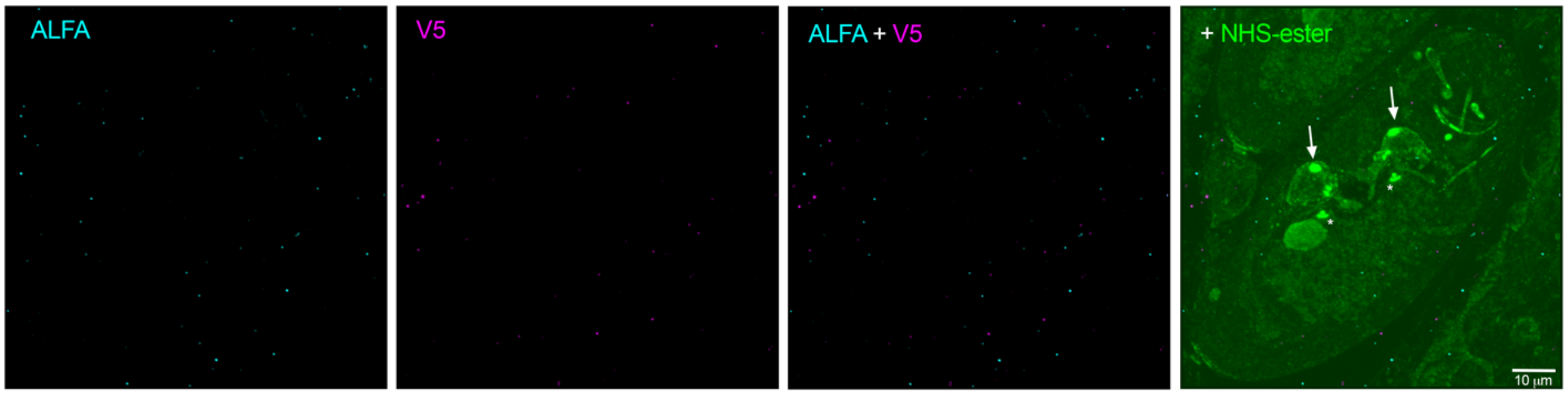

Antiserum negative control. Pan-ExM using RHΔKu80-Tir1 parasites. Gels were stained with V5 (magenta) and ALFA (cyan) antiserum. NHS-ester ATTO488 (green) highlights protein density. Arrows indicate forming daughter cells, asterisks the spindle poles.

- Please divide the supplementary figure using letters (A, B, C...) otherwise it is hard to follow.

Supplementary Figure panels are now labelled as suggested.

- Why was the 5'end chosen of APP1 chosen? Did you also try the 3 end?

We initially generated the APP1-cKD line in which the Ty-1 epitope tag is fused to the 5'-end of the APP1 ORF. To stay consistent with this orientation we chose 5'-end modification also for the ALFA-epitope tag (facilitating high-resolution imaging) as well as for the TurboID fusion. We have not tried fusing a tag to the 3'-end of the APP1 ORF, but would like to refer to the APP1 study done in Dr. Peter Bradley's lab in which 3'-end tagging of APP1 was done. Importantly, both studies (Mead et al. and ours) come to the same conclusions.

- It is unclear when does the IMC3 recruitment happen. I think this should be mentioned in the figure and also in the figure 2b schematic.

We have highlighted IMC3 recruitment to the forming daughter cell scaffolds in Figure 2b and the corresponding figure legend.

- The authors should choose a different title for figure 3 ("APP1 depletion disrupts apicoplast partitioning") as the authors don't examine apicoplast partitioning in this figure.

Thanks for pointing out this oversight. We changed the title to "APP1 is critical for proliferation, but does not affect replication rate or partitioning of the centrosome, Golgi apparatus, and mitochondrion".

- Figure 4b does not state how many vacuoles were counted.

We regret this oversight and have added the number of counted vacuoles (100 vacuoles for n=3 biological replicates) to the legend.

Then, in figure 4c, the authors measure apicoplast loss with qPCR. The control strain used is the parental strain (TATiΔHXGPRT), but shouldn't the authors use the conditional knockdown strain minus ATc as a control? If not, can the authors explain why? In addition, why did the authors express their qPCR data as fold as % of vacuoles rather than fold expression?

We apologize for mislabeling the graph in Figure 4C. The label has been corrected to "% DNA ratio (apicoplast/nucleus)". We followed established protocols for this analysis (PMID:

29470517) and quantified the copies of the apicoplast genome and the nuclear genome on day 0 (in absence of ATc) for the APP1-cKD as well as for the parental (TATiΔHX) strain. Parasites were then treated for 72 hrs with ATc and the copies again determined for both strains. The fold change of the apicoplast genome was then calculated for each individual line (using day 0 and day 3 samples) via the $2^{-\Delta\Delta CT}$ method, which indicates a 41% reduction (0.58-fold) of the apicoplast genome within the APP1-cKD strain after ATc treatment (data can be found in the Source file). This is in line with data presented in Figure 4a and b, which indicate that many but not all APP1-cKD parasites lose the apicoplast upon ATc treatment in the given time frames. To set this in relation to the parental strain, we set the $2^{-\Delta\Delta CT}$ value of TATiΔHX to 100% and calculated the reduction observed in APP1-cKD. We have clarified this in the result section (lines 169-172).

- Is there an explanation to why arbitrary units were chosen to quantify the plaque assays? Just a brief explanation will suffice.

We did not calibrate the pixel size of the scanner.

- There are no arrows in figures 4d, 5b and 5c even though they are indicated in the main manuscript.

We apologize for this oversight. We have added the missing arrows to Figures 4 and 5.

- The title for Figure 7 reads, “APP3 is critical for proliferation and localizes to the outer core of the centrosome prior to APP” – the authors should change APP to APP1. In addition, there is a typo for Figure 7f – APP3 is listed twice in the fluorescent channel legend.

We have made the suggested changes in legend and figure.

Reviewer #2 (Remarks to the Author):
I co-reviewed this manuscript with one of the reviewers who provided the listed reports. This is part of the Nature Communications initiative to facilitate training in peer review and to provide appropriate recognition for Early Career Researchers who co-review manuscripts.

We support the involvement of early-career researchers and sincerely appreciate the time and effort the reviewer dedicated to evaluating our manuscript.

Reviewer #3 (Remarks to the Author):
I co-reviewed this manuscript with one of the reviewers who provided the listed reports. This is part of the Nature Communications initiative to facilitate training in peer review and to provide appropriate recognition for Early Career Researchers who co-review manuscripts.

We support the involvement of early-career researchers and sincerely appreciate the time and effort the reviewer dedicated to evaluating our manuscript.

Reviewer #4 (Remarks to the Author):
This is manuscript describes a very comprehensive characterization of 2 proteins critical for the division of the Toxoplasma apicoplast. The data presented is significant and substantially expands our understanding of the cell biology of this important parasite. There are only a few minor issues that can be addressed before publication.

We thank the reviewer for highlighting the significance of our study.

1. There is a lack of clarity as to the knockdown system used for APP3 depletion. For figure 7 they use the TATi system to test propagation in tissue culture. For figure 8, they switch to the AID system, but do not explain why or show that the AID system recapitulates the plaque phenotype seen in figure 7. More concerning is that in figure 8, some panels (A, F, B and D) use the mAID system other panels use the TATi system (+/- ATc). This needs to be clarified. Ideally, only one knockdown system per figure is presented. Also, for the mAID system, the authors should perform a time course in the presence of IAA to confirm that the protein levels are being depleted and when.

We regret the erroneous labeling in Figures 7&8, as only one parasite line was created for APP3. In this APP3-cKD line the 3'-end of the APP3 ORF was tagged with a mAID-5xV5 tag in the background of the Tir1-ALFA-APP1 strain. We have removed all referral to the TATi system in the respective figures.

Regarding the time course of APP3-cKD: We were unsuccessful in visualizing full length APP3 on western blot, which is a 4037 aa protein with a predicted molecular weight of 438 kDa. However, we have applied IAA in a time course experiment and quantified 50 dividing vacuoles (judged by presence of IMC3 positive daughter buds) in three biological replicates with visible APP3-V5 signal (done with the established APP3-cKD line) via IFA/microscopy (see Figure below). This showed that even 1 hr of IAA treatment is sufficient to deplete APP3 below the detection limit.

However, for the shorter IAA treatment periods, we cannot exclude that division starts (in particular APP1 positioning and onset of apicoplast partitioning) while APP3 is still present. We therefore chose the 24 hrs time point in our experimental design to confidently examine parasites that started division in absence of APP3.

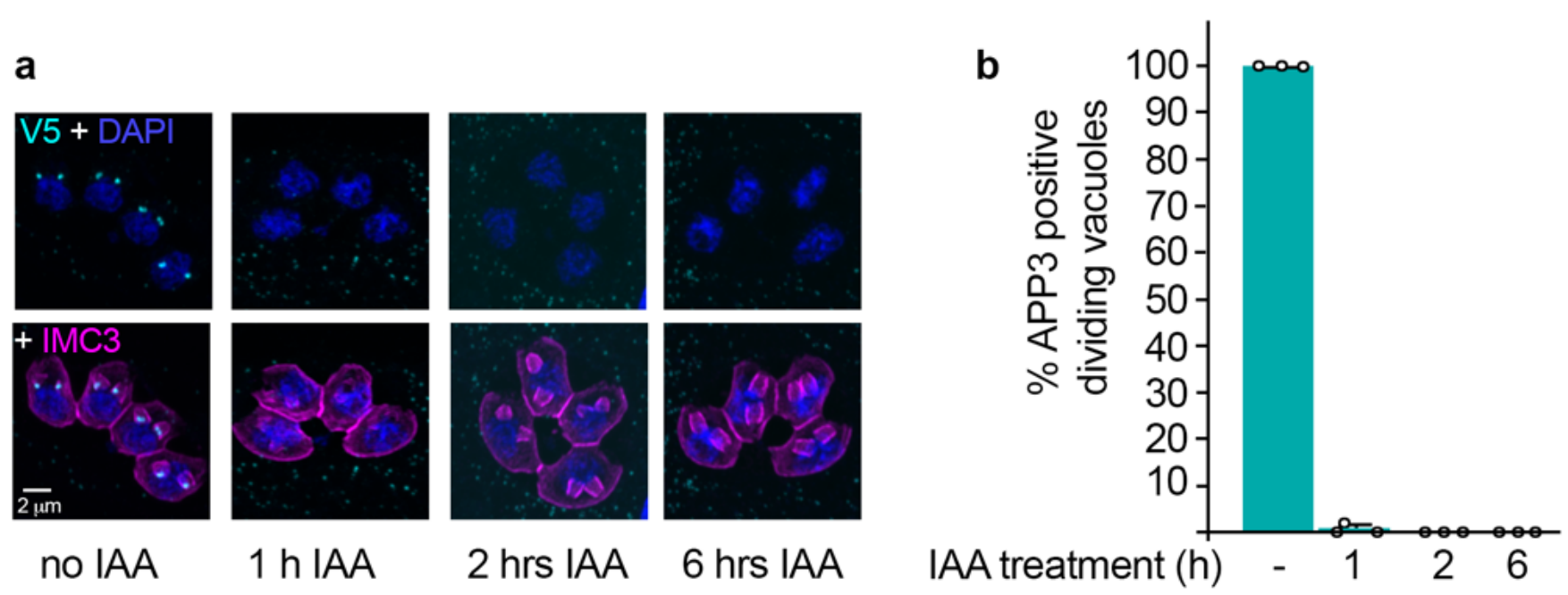

APP3 depletion timing. a). Representative images of APP3-cKD IAA treatment time course. Parasites were grown overnight and treated with IAA for indicated time periods at the next day. V5 antiserum recognizes APP3-mAID-V5 (APP3-cKD), DAPI stains DNA. **b).** Quantification of APP3 positive vacuoles. 50 dividing vacuoles in n=3 biological replicates were examined via IFA for APP3 presence. APP3 falls under the detection limit within 1 h of IAA treatment.

2. Figure 7F: APP3 is labeled twice.

We have corrected the double labeling. This IFA examines positioning of APP1 in relation to APP3.

3. Figure 8: Authors should show the staining for both ALPHA-APP1 and APP3-mAID-5xV5 to confirm dual tagging.

As a result of the mislabeling in Figure 7F (see #2 above), this point was unfortunately not communicated clearly: this exact dual labeling is shown in Figure 7F, resolved through steps in budding.

4. The summaries for each of the result sessions is a great feature of the paper, but at times it goes beyond summarizing and moves into speculations that are not directly supported by the data in the particular session. It might be better to limit the summaries to a recap of the results and moving the speculation and interpretation to the discussion session.

We have edited each result summary to be in line with the provided data of the respective paragraph, hereby omitting speculations and interpretations that are beyond the presented data.

5. The manuscript by Mead et al does a very good job of referring to this manuscript and discussing similarities and differences. This manuscript should do the same and draw parallels between the two studies.

This is a great suggestion and as such we have highlighted APP2 in our TurboID-APP1 figure (Figure 6a and lines 228-230). We further reference to the manuscript by Mead et al in lines 369-374.

Reviewer #5 (Remarks to the Author):
I co-reviewed this manuscript with one of the reviewers who provided the listed reports. This is part of the Nature Communications initiative to facilitate training in peer review and to provide appropriate recognition for Early Career Researchers who co-review manuscripts.

We support the involvement of early-career researchers and sincerely appreciate the time and effort the reviewer dedicated to evaluating our manuscript.

Reviewer #6 (Remarks to the Author):
Summary
In this study, Eichlin et al. identify and characterize TGGT1_287970 (APP1) and demonstrate that it functions as a connector between the apicoplast and the daughter cell cytoskeleton during Toxoplasma gondii endodyogeny. Using an elegant combination of genetic perturbations, advanced imaging approaches, and proximity biotinylation, the authors convincingly dissect the mechanistic role of APP1. In addition, they identify a second factor, APP3, and show that APP3 acts upstream to position APP1 at a specific subdomain of the basal complex. Together, these findings define a hierarchical system that links the apicoplast to the cell division machinery. This work therefore represents a conceptual advance, as it identifies a dedicated anchoring machinery underlying faithful apicoplast inheritance.

Own opinion
This is a very strong and well executed study that is important and suitable for publication in Nature Communications. The work addresses a long-standing question in apicomplexan cell biology, providing novel, mechanistic insights by identifying an anchoring apparatus, critical for apicoplast partitioning. A particular strength of the manuscript is the high quality of imaging. The localisation analysis is very beautiful and well executed. Overall the manuscript is very rigurous and my comments are only minor and can be adrdressed relatively easily.

We thank the reviewer for highlighting the importance of our findings.

Concerns:

1) Many of the key conclusions rely on representative images rather than quantitative analysis. However, since one central point of the study is the dynamic behaviour of APP1 and APP3, additional quantification would substantially strengthen the manuscript. In particular: The proximity of APP1 to the centrosome and apicoplast should be quantified by distance measurements. Similarly, the asymmetric localization of APP1 to the BC should be quantified.

We agree with the reviewer that microscopy data should be supported by measurements/quantifications whenever possible. We have followed this philosophy for the majority of experiments presented in our manuscript.
Regarding the distance between APP1 and the centrosome, data presented in Figure 2C indicate that both structures are in immediate proximity. The APP1 signal furthermore appears to engulf the centrosome, which does not make it immediately clear from where a measurement should be started. Similarly, the pan-ExM image in Figure 5C highlights the very close apposition of APP1 to the apicoplast membrane (highlighted via ATG8), to underline this we have used intensity profile measurements shown below.
To examine the asymmetric positioning of APP1 in the basal complex, we quantified its BC localization in presence or absence of APP3, these new data are now shown in Figure 8e.

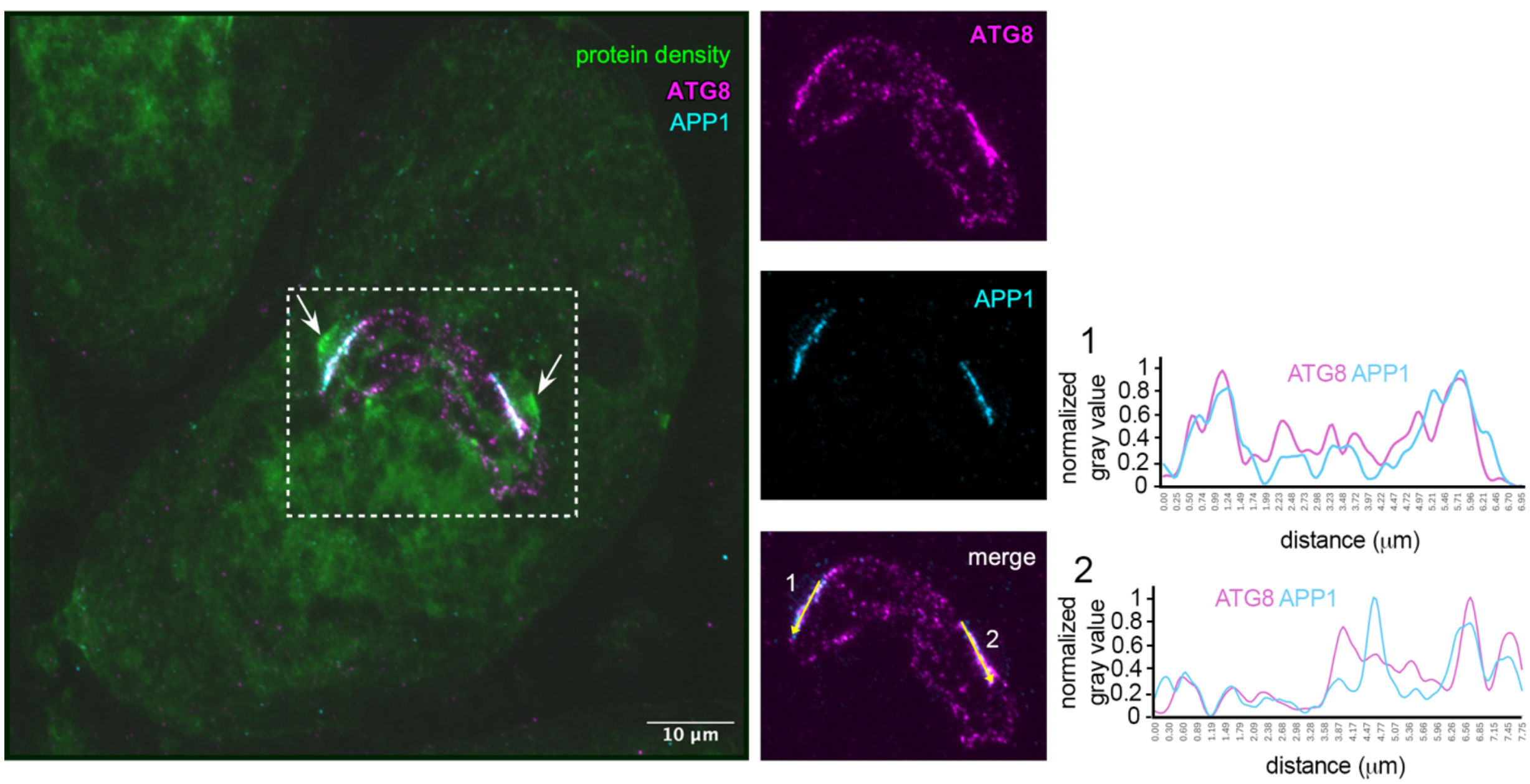

Intensity profiles of ATG8 and APP1. Pan-ExM (Fig 5c) indicates the close apposition of APP1 to the apicoplast outer membrane (highlighted via ATG8). Numbers in the "merge" image refer to the respective intensity prolife shown on the right. Yellow arrows indicate the measured area and direction. Gray values were recorded using FIJI and normalized using the min-Max method.

2) The proposed model relies on dynamic behaviour of APP1 and APP3 during the cell cycle. While the fixed-imaging is of very high quality, the authors could strengthen this aspect by performing live-cell imaging.

Although we do appreciate the addition of a more precise temporal dynamic resolution on APP1 vs APP3 appearance, it would come at the cost of spatial resolution. We want to emphasize that in the data as presented, we carefully calibrated APP1 and APP3 appearance

at the ultrastructural level with well-established markers for key spatiotemporal events in daughter scaffold assembly, like the centrosome, α-tubulin and IMC3. Hence, we do not expect that live imaging of APP1 vs APP3 would dramatically change the current interpretation, especially since our hierarchical data show that APP1 recruitment requires proper APP3 deposition. Together with the extensive amount of work needed to create dual fluorescent protein-tagged parasite lines, we decided not to pursue this suggestion.

3) Line 159: The sentence "we generated a conditional knock-down line by replacing APP1's promoter with an anhydrous tetracycline (ATc) regulatable TetO7sag4 promoter" could be improved for clarity. I suggest: "We generated a conditional knock-down using the Tet-Transactivation system (TATi)."

We have made the modification as suggested. .

4) The discussion appropriately addresses the limited phylogenetic conservation of APP proteins. Expanding this point slightly—particularly in comparison to systems such as PfAnchor in Plasmodium could further strengthen the evolutionary context of the study.

We appreciate the reviewer's comment, but want to respectfully point out that PfAnchor and the absence of any clear ortholog in *T. gondii* is discussed in our manuscript. We further speculate about the existence of a so far unidentified orthologous mechanism responsible for recruiting the apicoplast fission machinery in *T. gondii* (lines 364-368).

REVIEWERS' COMMENTS

Reviewer #1 (Remarks to the Author):

Thank you for the detailed response. I believe the authors have addressed all the comments from the reviewers, and the manuscript is ready for publication.

> We thank the reviewer for the positive assessment and for the constructive and helpful comments, which have contributed to improving our manuscript

Before that, I would like to suggest one minor modification: on figure 5b, the authors should use the same terminology describing endodyogeny as in figures 2 and 4: bud initiation, early budding, mid budding and late budding.

> This is a great suggestion, and we have changes figure 5b accordingly.

Reviewer #2 (Remarks to the Author):

I co-reviewed this manuscript with one of the reviewers who provided the listed reports. This is part of the Nature Communications initiative to facilitate training in peer review and to provide appropriate recognition for Early Career Researchers who co-review manuscripts.

> We support the involvement of early-career researchers in the peer-review process and thank the reviewer for their contribution to the evaluation of our manuscript.

Reviewer #3 (Remarks to the Author):

I co-reviewed this manuscript with one of the reviewers who provided the listed reports. This is part of the Nature Communications initiative to facilitate training in peer review and to provide appropriate recognition for Early Career Researchers who co-review manuscripts.

> We support the involvement of early-career researchers in the peer-review process and thank the reviewer for their contribution to the evaluation of our manuscript.

Reviewer #4 (Remarks to the Author):

The authors have responded to the reviews appropriately and have strengthened the manuscript in the process.

> We thank the reviewer for this positive assessment and appreciate their constructive and helpful comments.

Reviewer #5 (Remarks to the Author):

I co-reviewed this manuscript with one of the reviewers who provided the listed reports. This is part of the Nature Communications initiative to facilitate training in peer review and to provide appropriate recognition for Early Career Researchers who co-review manuscripts.

We support the involvement of early-career researchers in the peer-review process and thank the reviewer for their contribution to the evaluation of our manuscript.

Reviewer #6 (Remarks to the Author):

In their revision, the authors provided a detailed response to the reviewers comments and addressed all my concerns. I think the revised manuscript is significantly improved and provides a compelling and rigorous characterization of the machinery governing apicoplast inheritance in Toxoplasma gondii. I have no further comments and recommend the manuscript for publication and congratulate the authors to a very nice study.

We sincerely thank the reviewer for the positive assessment of our revised manuscript and for the constructive comments provided throughout the review process, which helped us further strengthen the manuscript. We greatly appreciate the reviewer’s kind words about our study.
